# Supplementary material for: A selective and atom-economic rearrangement of uridine by cascade biocatalysis for production of pseudouridine
Source: Nat Commun. 2023 Apr 20;14:2261. doi: 10.1038/s41467-023-37942-7 (PMC10116470; doi:10.1038/s41467-023-37942-7)
Supplement: Supplementary file 1 — Supplementary Information [file 41467_2023_37942_MOESM1_ESM.pdf]

Supplementary Information

A selective and atom-economic rearrangement of uridine by cascade biocatalysis for production of pseudouridine

Martin Pfeiffer[1,2], Andrej Ribar[1,2] and Bernd Nidetzky[1,2,*]

[1]Institute of Biotechnology and Biochemical Engineering, Graz University of Technology, NAWI Graz, Petersgasse 12, A-8010 Graz, Austria

[2]Austrian Centre of Industrial Biotechnology (acib), Krenngasse 37, A-8010 Graz, Austria

* Correspondence to B.N., e-mail: bernd.nidetzky@tugraz.at

Supplementary Figure 1. Michaelis Menten plots for **a-b**) UP, **c-d**) YeiN, **e**) DeoB and **f-g**) Yjjg 4
Supplementary Figure 2. Inhibition of DeoB by phosphate. 5
Supplementary Figure 3. Enzyme melting curves determined by differential scanning fluorometry.... 6
Supplementary Figure 4. Enzyme inactivation under operational conditions. 7
Supplementary Figure 5. SDS polyacrylamide gel showing result of typical enzyme preparations. 8
Supplementary Figure 6. Soluble uracil present in a YeiN catalyzed synthesis of ΨMP starting from 0.10 M uracil. 9
Supplementary Figure 7. The influence of (**a**) UP, (**b**) DeoB and (**c**) YeiN concentration on the ΨMP-formation rate and conversion in the three-enzyme cascade under standard conditions. 10
Supplementary Figure 8. The influence of (**a**) pH, (**b**) temperature, (**c**) $MnCl_2$ and (**d**) phosphate on the ΨMP-formation rate and conversion in the three-enzyme cascade under standard conditions. 11
Supplementary Figure 9. Addition of $MnCl_2$ controls the precipitation of $Mn_3(PO_4)_2$ or uracil. 12
Supplementary Figure 10. Product intensification of the three-enzyme cascade under improved reaction conditions. 13
Supplementary Figure 11. Time courses of three-enzyme cascade reactions with reduced enzyme loading. 14
Supplementary Figure 12. Effect of Glc1,6diP on the three-enzyme cascade reaction under standard conditions. 15
Supplementary Figure 13. Reaction time course of ΨMP synthesis at 5 mL (solid line) and 100 µL (dashed line) scale. 16
Supplementary Figure 14. ^{1}H NMR of ΨMP. 17
Supplementary Figure 15. ^{13}C NMR of ΨMP. 17
Supplementary Figure 16. ^{31}P NMR of ΨMP. 18
Supplementary Figure 17. COSY NMR of ΨMP. 19
Supplementary Figure 18. HSQC NMR of ΨMP. 19
Supplementary Figure 19. HMBC NMR of ΨMP. 20
Supplementary Figure 20. HPLC trace of isolated ΨMP. 20
Supplementary Figure 21. Synthesis of ΨTP. 21
Supplementary Figure 22. ^{1}H NMR of ΨTP 22
Supplementary Figure 23. ^{13}C NMR of ΨTP 22
Supplementary Figure 24. ^{31}P NMR of ΨTP 23
Supplementary Figure 25. HPLC trace of isolated ΨTP. 23
Supplementary Figure 26. The effect of phosphate concentration on the (**a**) Ψ formation rate, conversion and (**b**) ΨMP steady state concentration in the four-enzyme cascade reaction under standard conditions. 24
Supplementary Figure 27. Effect of Yjjg loading on the (**a**) Ψ formation rate, conversion and (**b**) ΨMP steady state concentration in the four-enzyme cascade reaction. 24
Supplementary Figure 28. The effect of (**a**) $MnCl_2$ concentration and (**b**) temperature on the Ψ formation and conversion in the four-enzyme cascade. 25
Supplementary Figure 29. Product intensification in the four-enzyme cascade reaction at optimized reaction conditions.. 26
Supplementary Figure 30. Time courses of the four-enzyme cascade reaction with reduced enzyme loading. 27
Supplementary Figure 31. Scalability of the four-enzyme cascade reaction. 28
Supplementary Figure 32. ^{1}H NMR of Ψ. 29
Supplementary Figure 33. ^{13}C NMR of Ψ. 29
Supplementary Figure 34. ^{31}P NMR of Ψ. 30
Supplementary Figure 35. COSY-NMR of Ψ. 30

Supplementary Figure 36. HSQC-NMR of Ψ. 31
Supplementary Figure 37. HMBC-NMR of Ψ. 31
Supplementary Figure 38. HPLC trace of isolated Ψ.. 32
Supplementary Figure 39. Synthesis of Ψ from UMP using the four-enzyme cascade reaction. 33
Supplementary Figure 40. HPLC trace of authentic reference substances used and their separation. 34
Supplementary Figure 41. HPLC trace of method used for analysis of phosphorylation reactions. 34
Supplementary Figure 42. ^{1}H NMR of Rib1P 35

Supplementary Table 1. Solubility of Ψ, U, and Ura in water.. 9

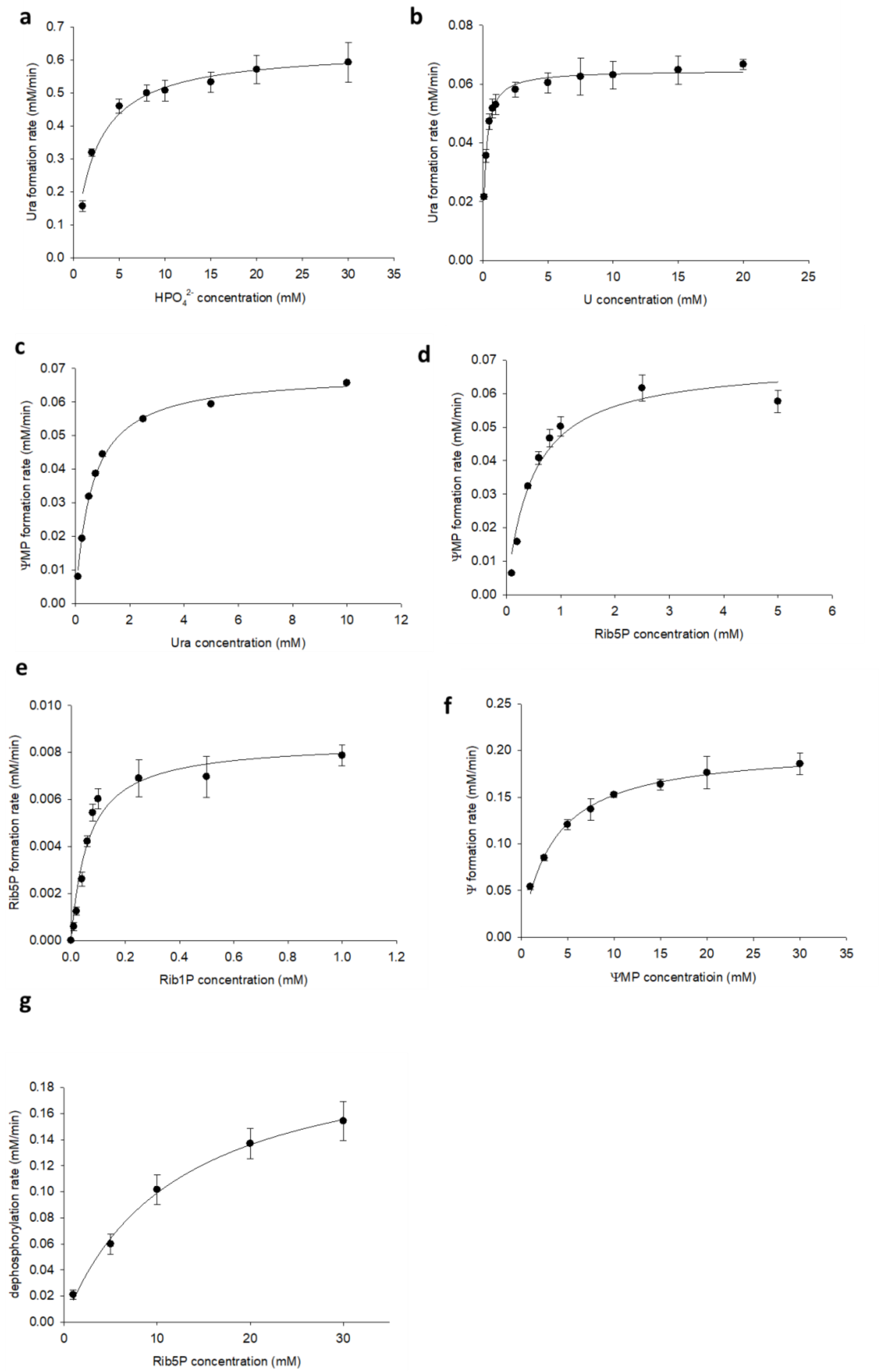

Supplementary Figure 1. Michaelis Menten plots for **a-b**) UP, **c-d**) YeiN, **e**) DeoB and **f-g**) Yjjg. Initial rates were determined in 50 mM HEPES buffer (pH 7.0) containing 2.0 mM $MnCl_2$ at 30°C (n = 3 individual experiments). Mean values are shown with S.D. indicated by error bars. The solid lines are fits of the data with Equation 1 (main manuscript). For further experimental details and for the analytical procedures used, see the Methods section of the main manuscript. Source data are provided as a Source Data file.

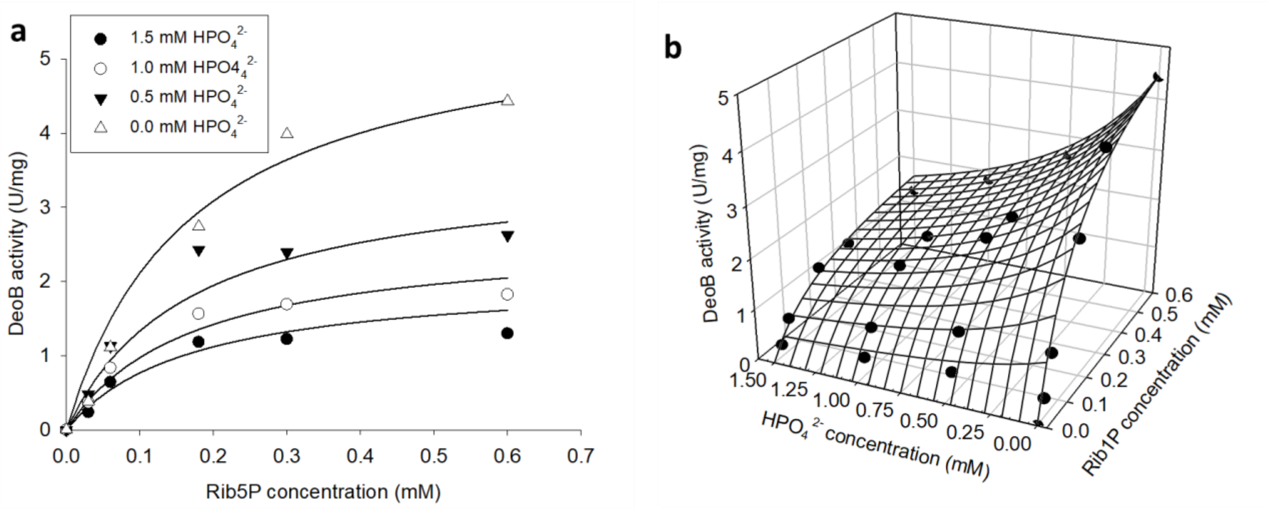

Supplementary Figure 2. Inhibition of DeoB by phosphate. **a**) Michaelis Menten plot showing noncompetitive inhibition. The K_M is unaffected while the V_{max} decreases with increasing phosphate concentration. Global fit of the data to the equation for noncompetitive inhibition (Equation 2 of main manuscript) is shown with solid lines. **b**) Three-dimensional representation of the data with fit results superimposed as line grid. Initial rates were determined in 50 mM HEPES buffer (pH 7.0) containing 2.0 mM $MnCl_2$ at 30°C (n = 1 individual experiments). For further experimental details and for the analytical procedures used, see the Methods section of the main manuscript. Source data are provided as a Source Data file.

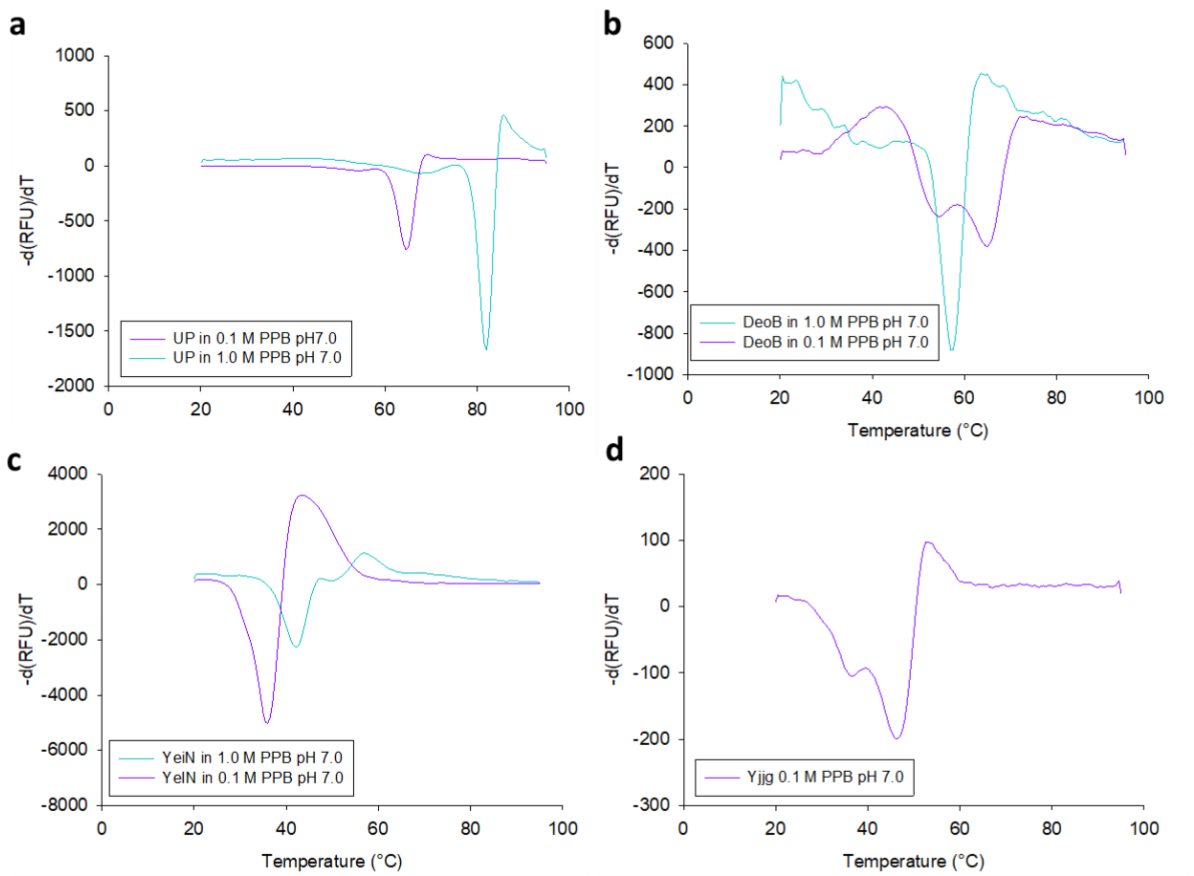

Supplementary Figure 3. Enzyme melting curves determined by differential scanning fluorometry. **a**) UP, **b**) DeoB, **c**) YeiN and **d**) Yjjg. The temperature at which the derivative is the lowest (most negative) is taken as the melting temperature (T_m). The thermal shift assay was performed in 0.1 M or 1.0 M potassium phosphate buffer (pH 7.0). The plots show the average melting curve of three independent measurements, subtracted by the buffer control. For detailed conditions, see the Methods section of the main manuscript. The enzyme concentration used was ~5 µM. Source data are provided as a Source Data file.

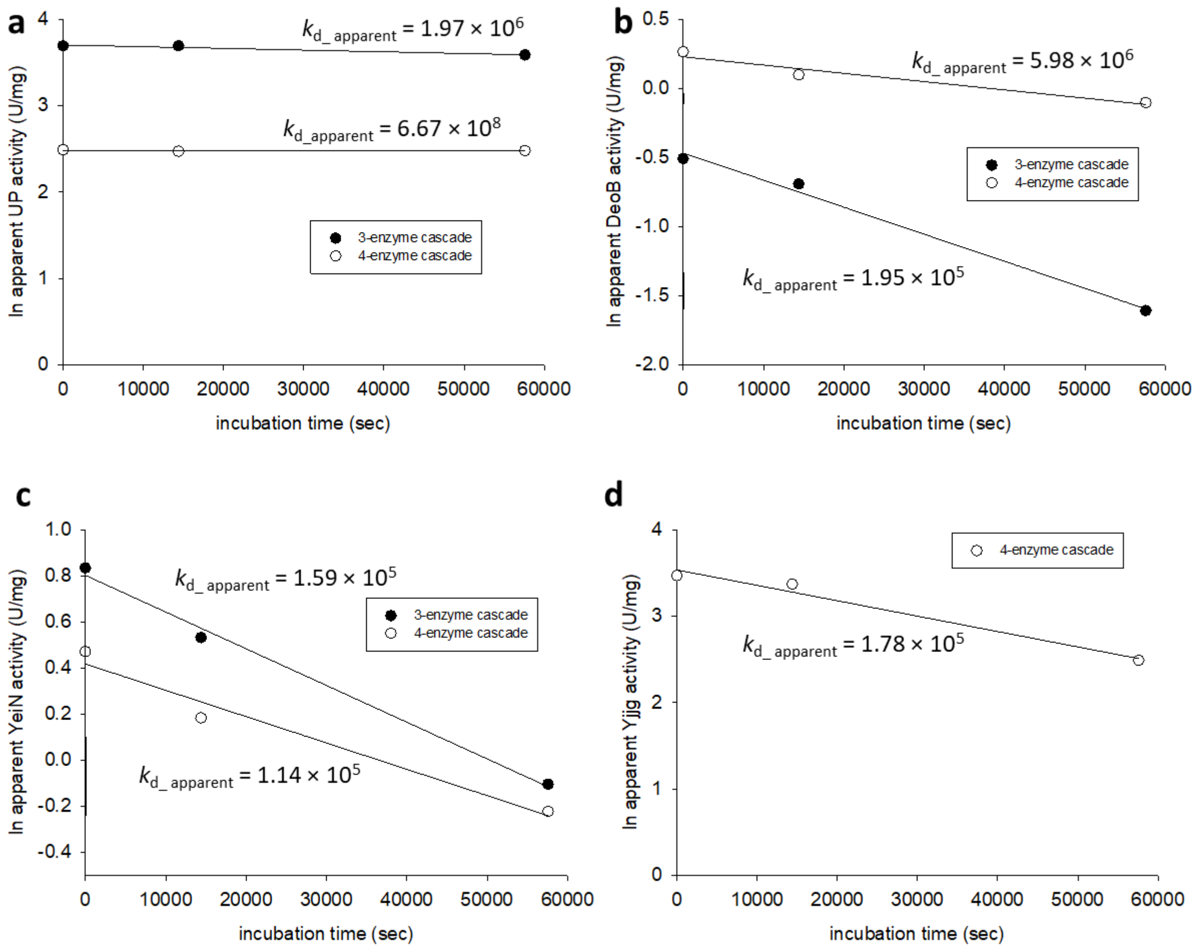

Supplementary Figure 4. Enzyme inactivation under operational conditions. **a**) UP, **b**) DeoB, **c**) YeiN, **d**) Yjjg. Individual enzymes were incubated in 1.0 M potassium phosphate buffer (pH 7.0) containing 20 mM $MnCl_2$ at 40°C or in 0.1 M potassium phosphate buffer (pH 7.0) containing 2.5 mM $MnCl_2$ at 30 °C. The conditions mimic the environment of the three- or four-enzyme cascade reaction, respectively. Residual enzyme activity was measured in samples after 4 h and 16 h, using the enzyme-specific assays. (n = 1 individual experiment) For further experimental details and for the analytical procedures used, see the Methods section of the main manuscript. The lines show linear fits of the data, with slope values given (k_d_apparent). Source data are provided as a Source Data file.

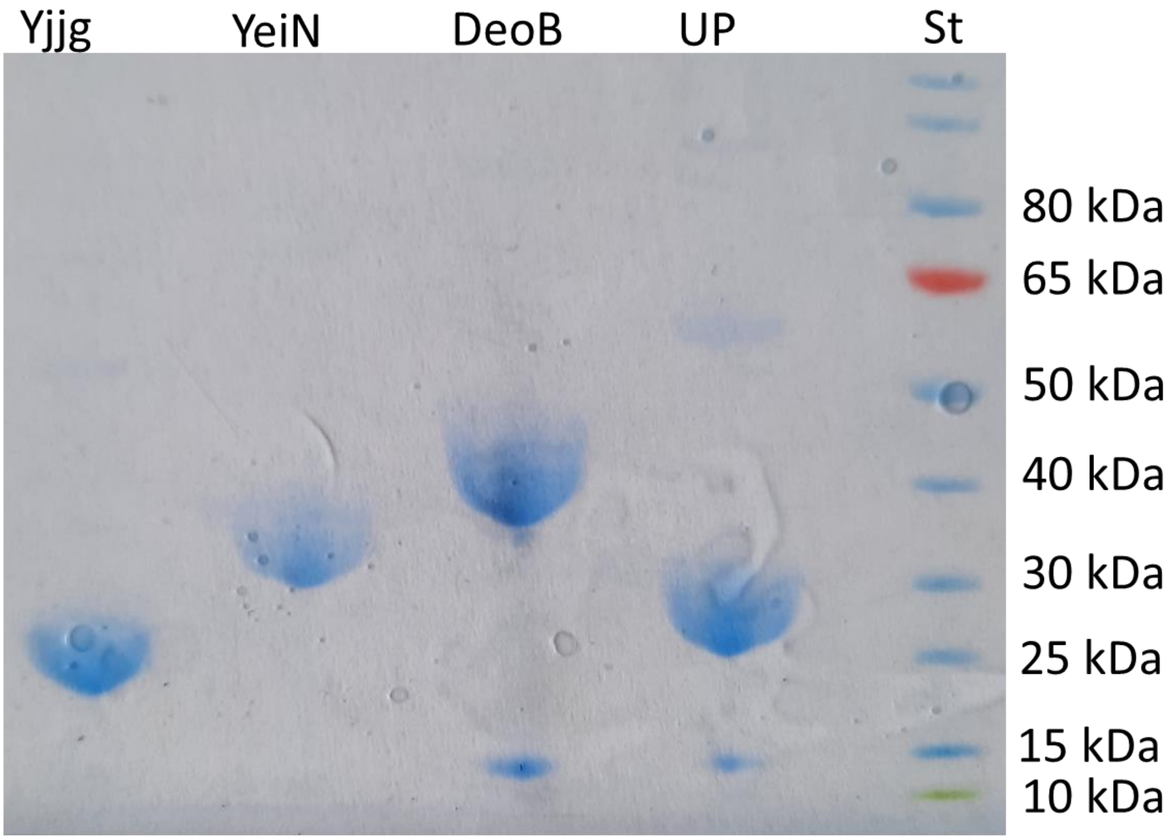

Supplementary Figure 5. SDS polyacrylamide gel showing result of typical enzyme preparations. Yjjg: nucleoside monophosphate phosphatase, YeiN: β-pseudouridine 5'-phosphate glycosidase, DeoB: phosphopentosemutase, UP: uridine phosphorylase. St: protein size standard. Source data are provided as a Source Data file.

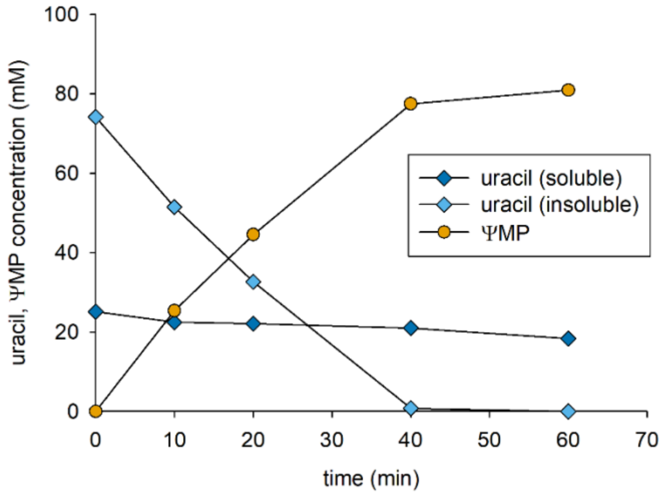

Supplementary Figure 6. Soluble uracil present in a YeiN catalyzed synthesis of ΨMP starting from 0.10 M uracil. Incubation was at 37 °C, pH 7.0 (water), and 900 rpm shaking. Rib5P and $MnCl_2$ were used at 0.15 M and 10 mM, respectively. The reaction was started with 0.2 mg/mL YeiN. For further experimental details and for the analytical procedures used, see the Methods section. Source data are provided as a Source Data file. (n = 1 individual experiment)

Supplementary Table 1.Solubility of Ψ, **U**, and Ura in water

Temperature (°C)	Ψ (mM)	U (mM)	Ura (mM)
30	200	1900	23
35	225	> 2000	26
40	260	> 2000	29
45	290	> 2000	33
50	380	> 2000	36

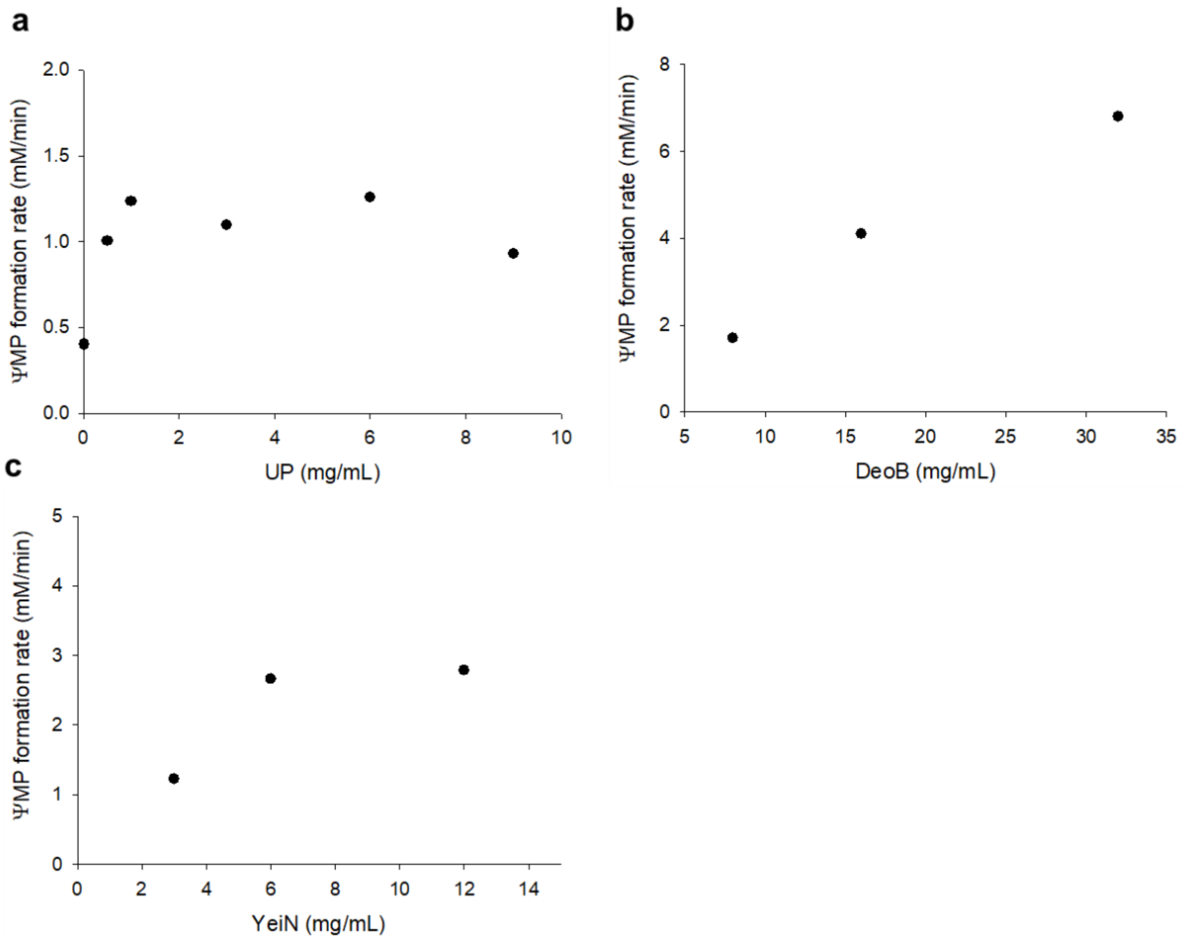

Supplementary Figure 7. The influence of (**a**) UP, (**b**) DeoB and (**c**) YeiN concentration on the ΨMP-formation rate and conversion in the three-enzyme cascade under standard conditions. Reactions were performed under standard conditions: 1.50 M potassium phosphate buffer (pH 7.0), 1.00 M **U**, 10 mM $MnCl_2$, 1 mg/mL UP, 10 mg/mL DeoB and 6 mg/mL YeiN at 100 µL scale and incubated at 30 °C. (**a**) the UP concentration was varied from 0.1 to 9.0 mg/mL; (**b**) the DeoB concentration was varied from 8 mg/mL to 32 mg/mL; (**c**) the YeiN concentration was varied to 3 mg/mL, to 12 mg/mL. For further experimental details and for the analytical procedures used, see the Methods section. (n =1 individual experiment) Source data are provided as a Source Data file.

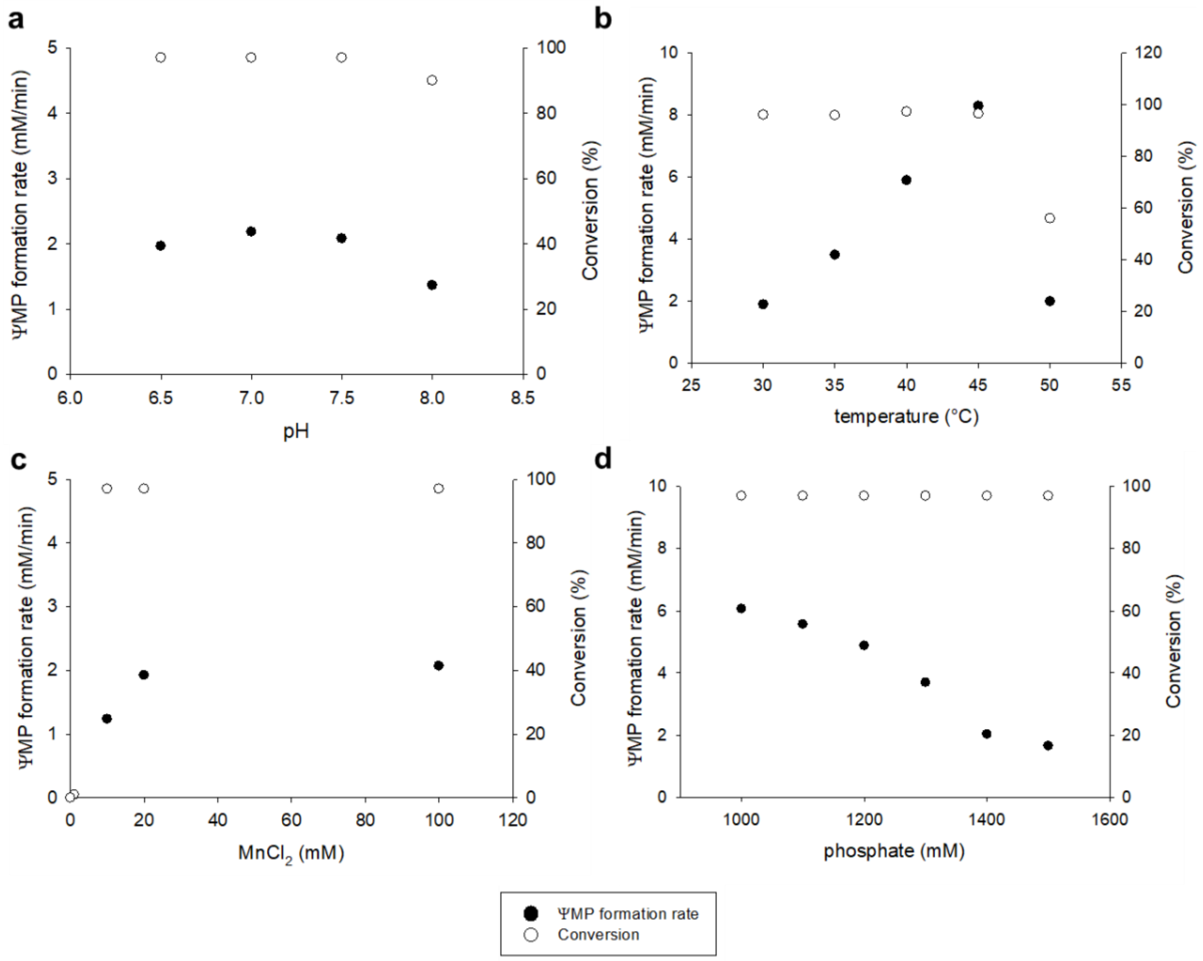

Supplementary Figure 8. The influence of (**a**) pH, (**b**) temperature, (**c**) $MnCl_2$ and (**d**) phosphate on the ΨMP-formation rate and conversion in the three-enzyme cascade under standard conditions. Reactions were performed under standard conditions: 1.50 M potassium phosphate buffer (pH 7.0), 1.00 M **U**, 10 mM $MnCl_2$, 1 mg/mL UP, 10 mg/mL DeoB and 6 mg/mL YeiN at 100 µL scale and incubated at 30 °C (n =1 individual experiment). For further experimental details and for the analytical procedures used, see the Methods section. Source data are provided as a Source Data file.

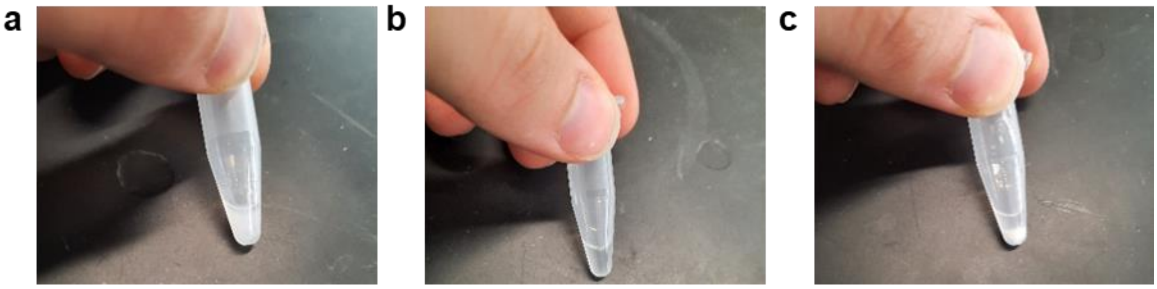

Supplementary Figure 9. Addition of $MnCl_2$ controls the precipitation of $Mn_3(PO_4)_2$ or uracil. The mixture after 180 min of reaction with (**a**) 100 mM, (**b**) 10 mM and (**c**) 1.0 mM of $MnCl_2$ under standard conditions is shown (n =1 individual experiment). Reaction conditions: 1.50 M potassium phosphate buffer (pH 7.0), 1.00 M **U**, 10 mM $MnCl_2$, 1 mg/mL UP, 10 mg/mL DeoB and 6 mg/mL YeiN, 30 °C. For further experimental details and for the analytical procedures used, see the Methods section.

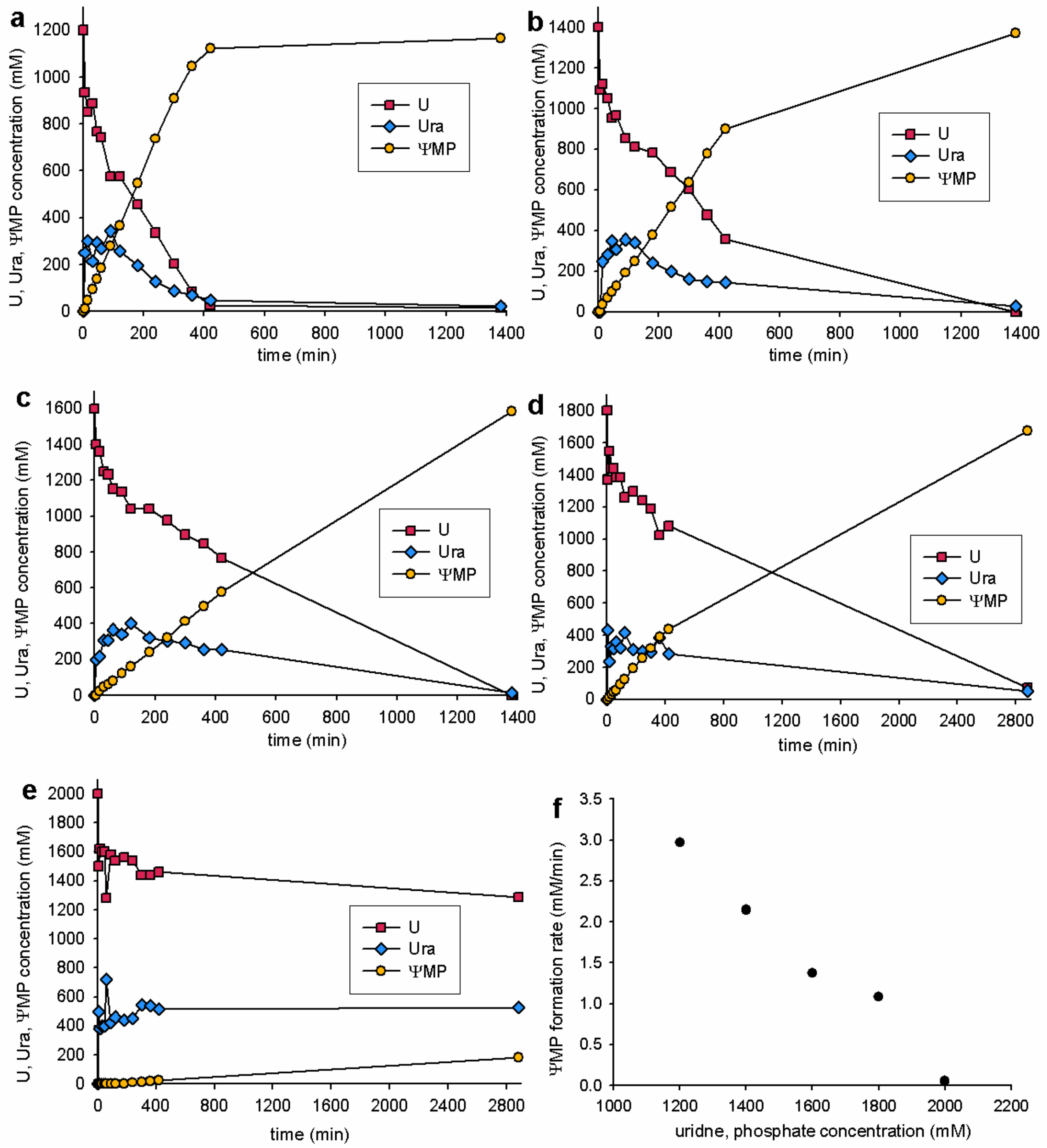

Supplementary Figure 10. Product intensification of the three-enzyme cascade under improved reaction conditions. (**a**) 1.20 M **U** and phosphate, (**b**) 1.40 M **U** and phosphate, (**c**) 1.60 mM **U** and phosphate, (**d**) 1.80 mM **U** and phosphate, (**e**) 2.00 mM **U** and phosphate, (**f**) effect of substrate concentration on the ΨMP formation rate. Reactions were performed using 1.20 M - 2.00 M **U** and an equimolar potassium phosphate buffer (pH 7.0) in respect to the **U** concentration, 20 mM $MnCl_2$, 1.0 mg/mL UP, 10 mg/mL DeoB and 6 mg/mL YeiN incubated at 40 °C (n =1 individual experiment). For further experimental details and for the analytical procedures used, see the Methods section. Source data are provided as a Source Data file.

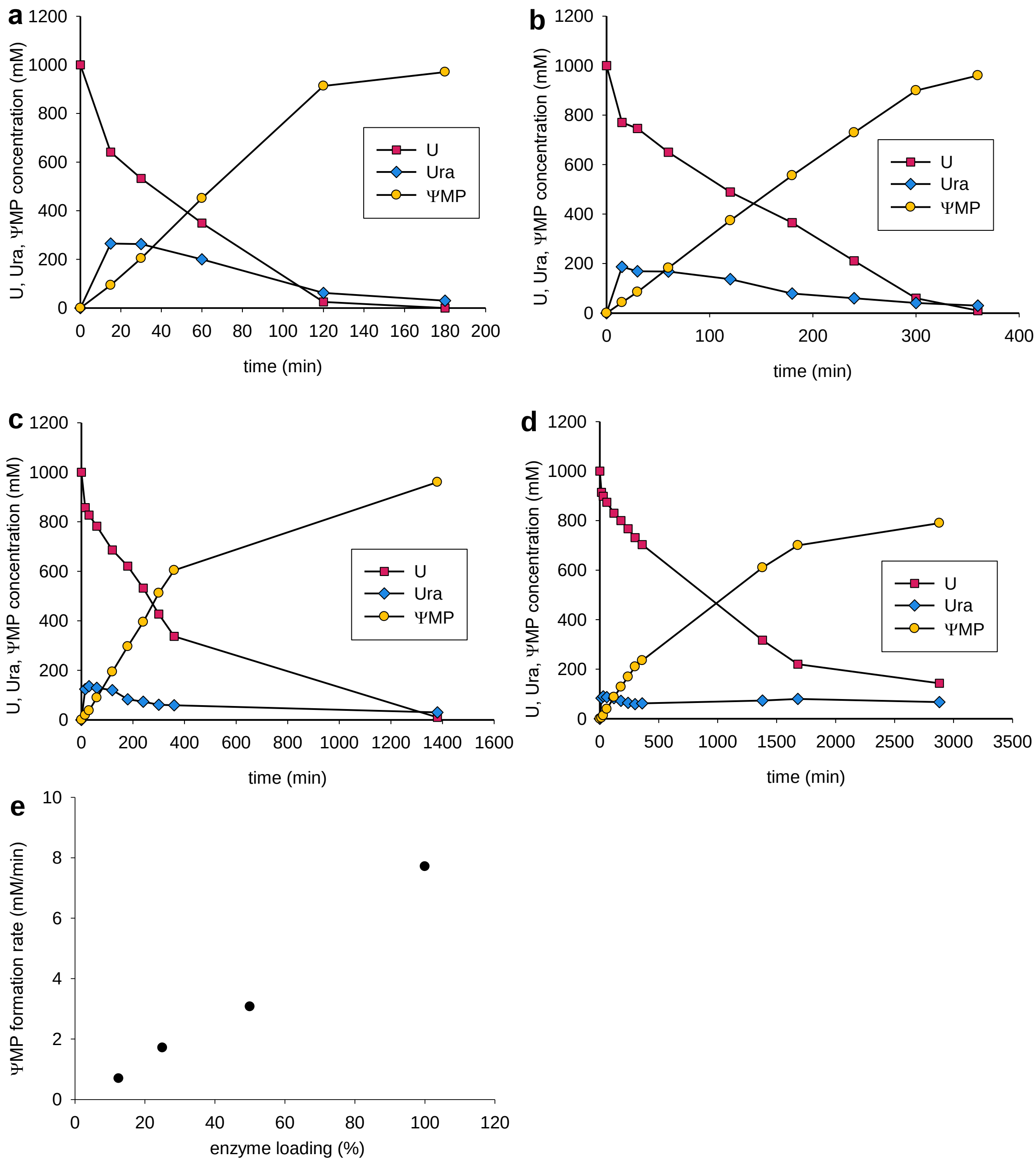

Supplementary Figure 11. Time courses of three-enzyme cascade reactions with reduced enzyme loading. Reactions were performed at 100 µL scale using 1.00 M **U** and an equimolar potassium phosphate buffer (pH 7.0), supplemented with 20 mM $MnCl_2$ incubated at 40 °C. (**a**) standard loading: 1 mg/mL UP, 10 mg/mL DeoB and 6 mg/mL YeiN, (**b**) two-fold reduced loading, (**c**) four-fold reduced loading, (**d**) eight-fold reduced loading (each n =1 individual experiment). (**e**) Effect of reduced enzyme loading on the **Ψ**MP formation rate. For further experimental details and for the analytical procedures used, see the Methods section. Source data are provided as a Source Data file.

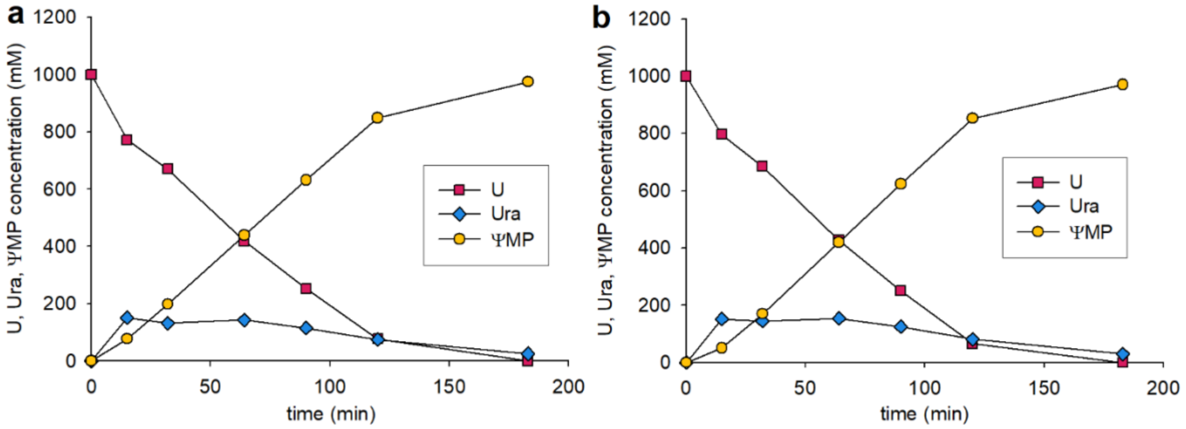

Supplementary Figure 12. Effect of Glc1,6diP on the three-enzyme cascade reaction under standard conditions. (**a**) Time course of the reaction without Glc1,6diP and (**b**) with addition of 0.1 mM Glc1,6diP. Reactions were performed using 1.00 M **U** and an equimolar potassium phosphate buffer (pH 7.0), supplemented with 20 mM $MnCl_2$, at 100 µL scale and incubated at 40 °C. **Ψ**MP synthesis was started with 1.0 mg/ mL UP, 10.0 mg/mL DeoB and 6.0 mg/mL YeiN. Source data are provided as a Source Data file. (n =1 individual experiment)

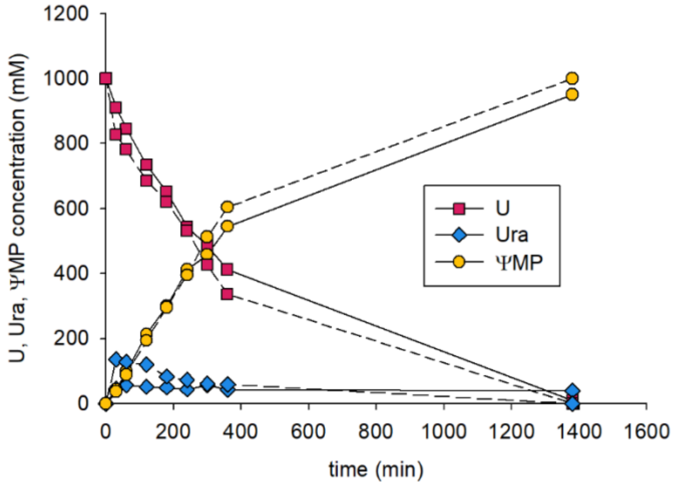

Supplementary Figure 13. Reaction time course of ΨMP synthesis at 5 mL (solid line) and 100 µL (dashed line) scale. 1.00 M potassium phosphate buffer (pH 7.0), 1.00 M **U**, 20 mM $MnCl_2$, 0.25 mg/mL UP, 2.5 mg/mL DeoB and 1.5 mg/mL YeiN were incubated at 40 °C. Source data are provided as a Source Data file. (n =1 individual experiment)

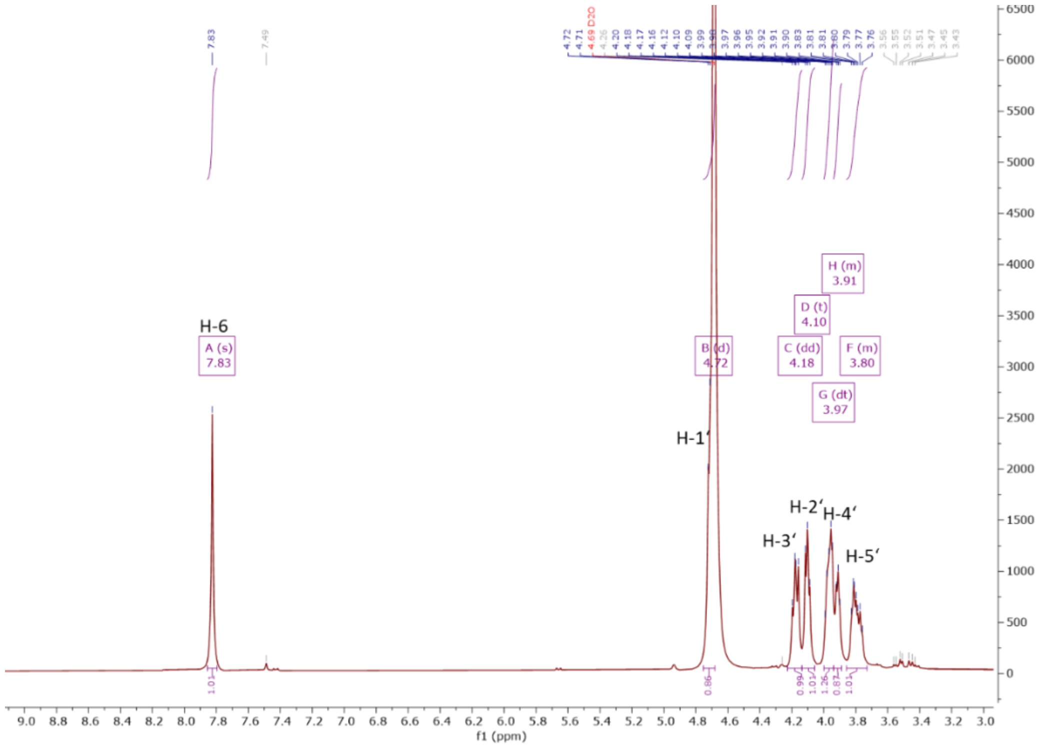

Supplementary Figure 14. ^{1}H NMR of ΨMP. (300 MHz, D_2O): δ 7.83 (s, 1H, H-6), 4.72 (d, *J* = 3.7 Hz, 1H, H-1'), 4.18 (dd, *J* = 6.7, 4.8 Hz, 1H, H-3'), 4.10 (t, *J* = 4.3 Hz, 1H, H-2'), 4.02 – 3.87 (m, 2H), 3.86 – 3.73 (m, 1H).

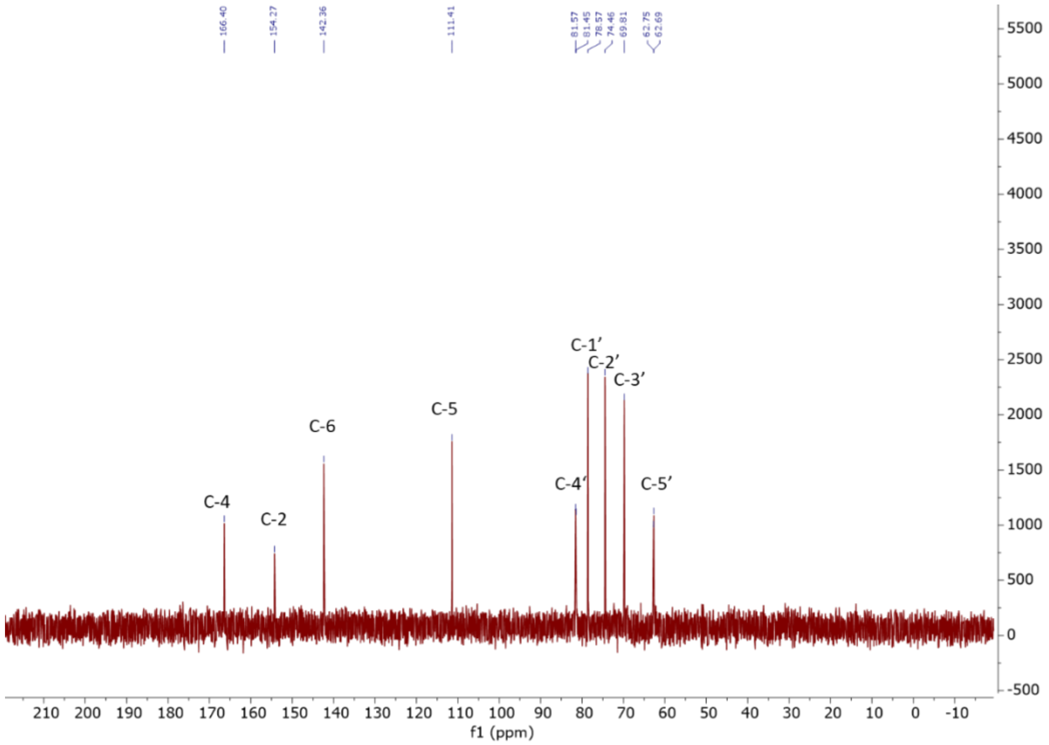

Supplementary Figure 15. ^{13}C NMR of ΨMP. (76 MHz, D_2O): δ 166.40 (C-2), 154.27 (C-4), 142.36 (C-6), 111.41 (C-5), 81.51 (d, *J* = 8.4 Hz) (C-4'), 78.57 (C-1'), 74.46 (C-2'), 69.81 (C-3'), 62.72 (d, *J* = 4.4 Hz) (C-5').

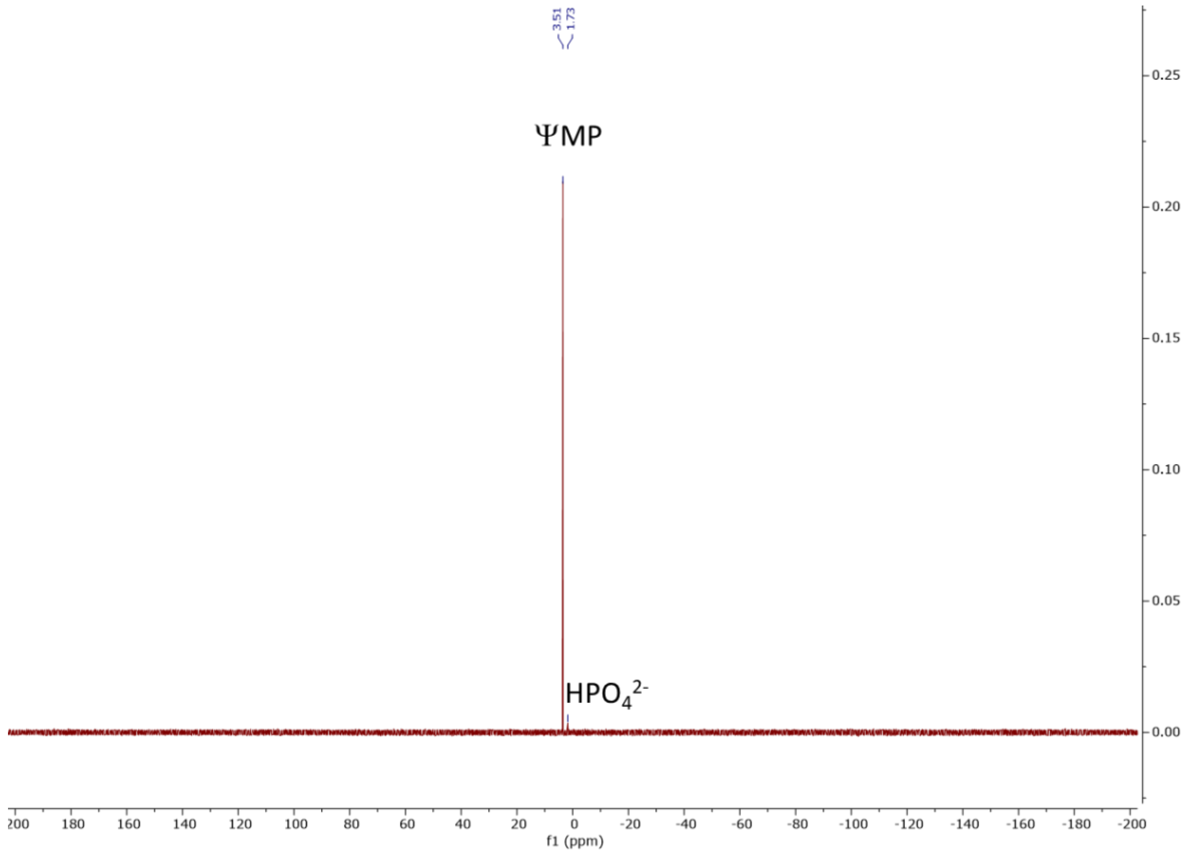

Supplementary Figure 16. ^{31}P NMR of ΨMP. (202 MHz, D_2O): δ 3.51 (ΨMP), 1.73 (HPO_4^{2-}). $MnCl_2$ was removed by treatment with Amberlite® IRC120 H.

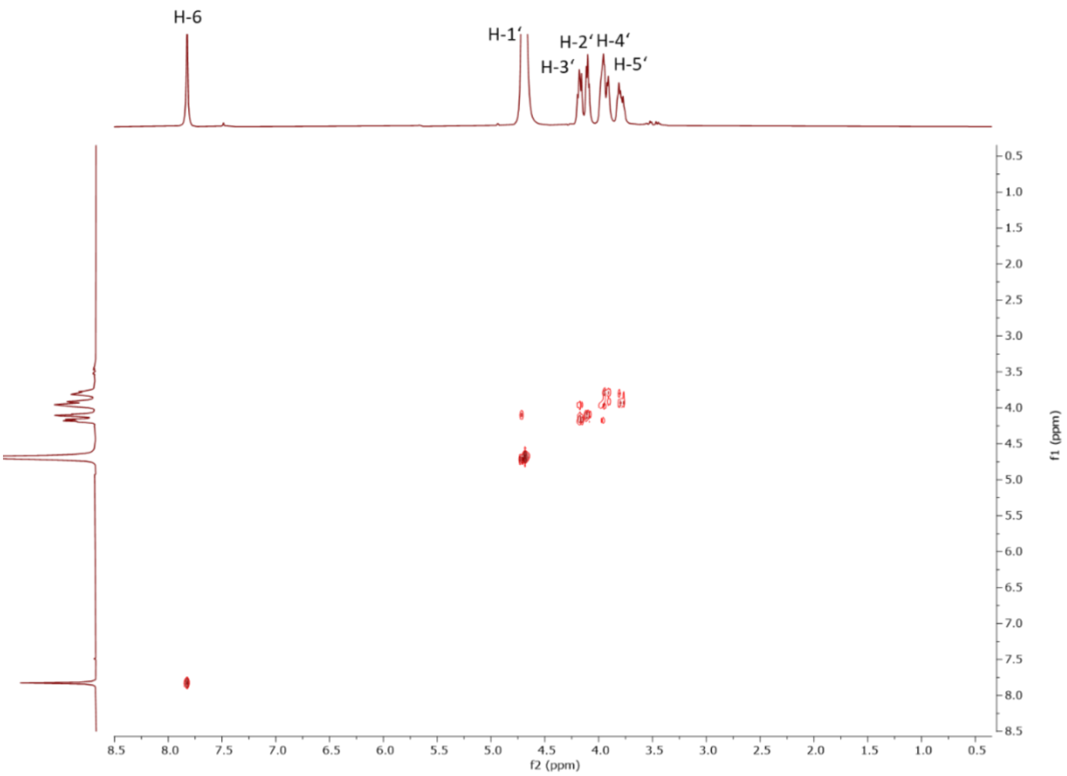

Supplementary Figure 17. COSY NMR of ΨMP. (300 MHz, D_2O): δ 7.83 (s, H-6, 1H), 4.69 (s, H-1', 1H), 4.18 (dd, *J* = 6.7, 4.8 Hz, H-3', 1H), 4.10 (t, *J* = 4.3 Hz, H-4', 1H), 3.97 (dt, *J* = 6.7, 2.9 Hz, 1H), 3.93 – 3.71 (m, H-5', 2H).

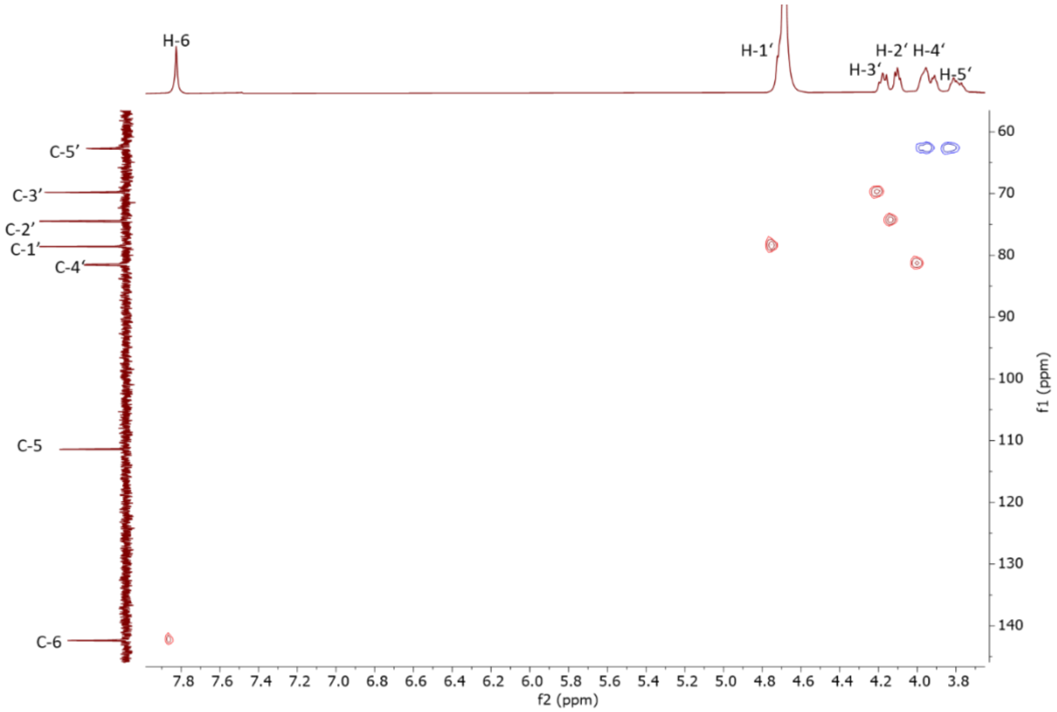

Supplementary Figure 18. HSQC NMR of ΨMP. ^{1}H NMR (300 MHz, D_2O): δ 7.83 (s, H-6, 1H), 4.69 (s, H-1', 1H), 4.18 (dd, *J* = 6.7, 4.8 Hz, H-3', 1H), 4.10 (t, *J* = 4.3 Hz, H-4', 1H), 3.97 (dt, *J* = 6.7, 2.9 Hz, 1H), 3.93 – 3.71 (m, , H-5', 2H); ^{13}C NMR (76 MHz, D_2O): δ 142.36 (C-6), 111.41 (C-5), 81.51 (C-4'), 78.57 (C-1'), 74.46 (C-2'), 69.81 (C-3'), 62.72 (C-5').

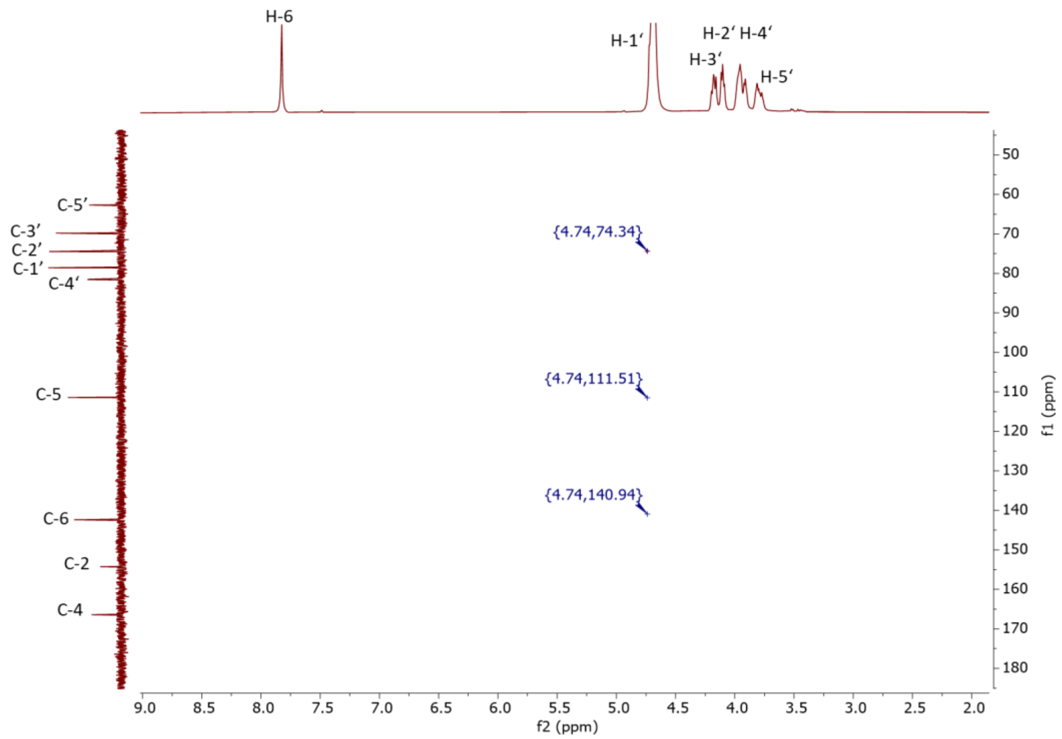

Supplementary Figure 19. HMBC NMR of ΨMP. ^1H NMR (300 MHz, D_2O): δ 7.83 (s, H-6, 1H), 4.69 (s, H-1', 1H), 4.18 (dd, *J* = 6.7, 4.8 Hz, H-3', 1H), 4.10 (t, *J* = 4.3 Hz, H-4', 1H), 3.97 (dt, *J* = 6.7, 2.9 Hz, 1H), 3.93 – 3.71 (m, , H-5', 2H); ^{13}C NMR (76 MHz, D_2O): δ 166.40 (C-4), 154.27 (C-2), 142.36 (C-6), 111.41 (C-5), 81.51 (C-4'), 78.57 (C-1'), 74.46 (C-2'), 69.81 (C-3'), 62.72 (C-5').

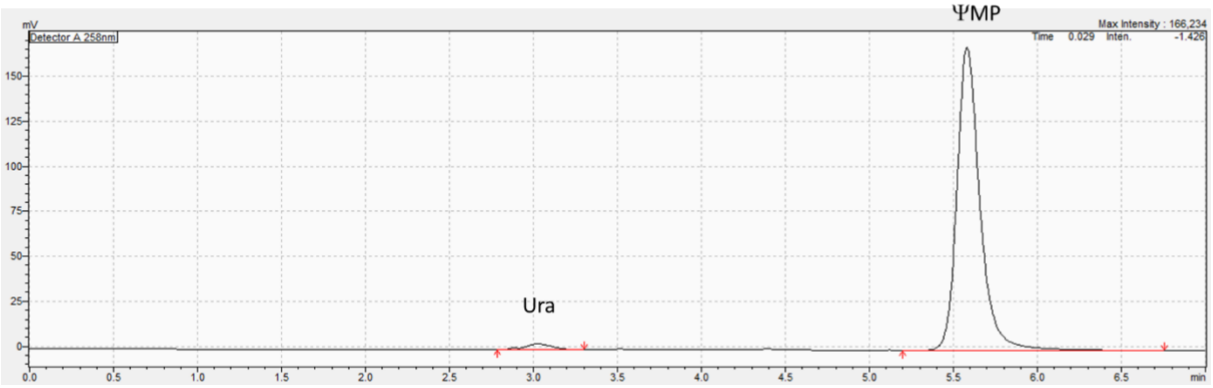

Supplementary Figure 20. HPLC trace of isolated ΨMP.

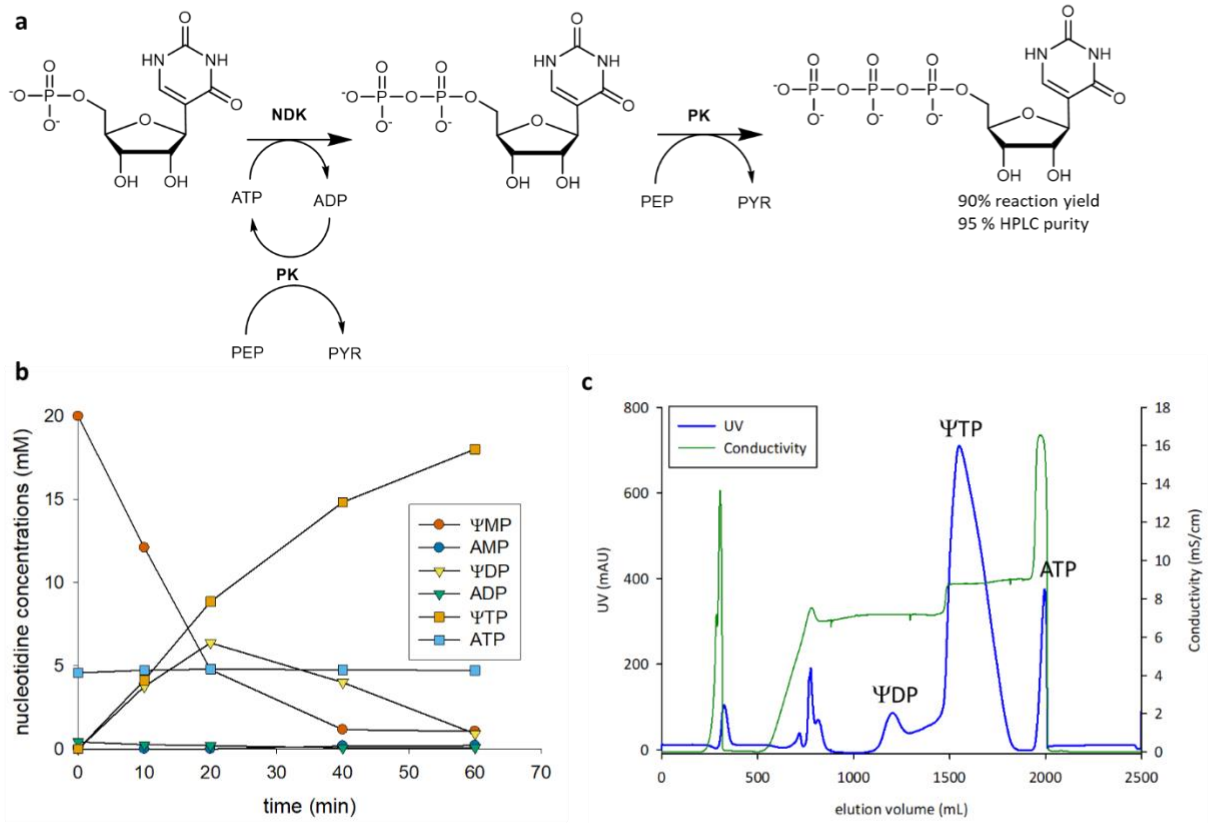

Supplementary Figure 21. Synthesis of ΨTP. **a**) shows the reaction scheme for ΨTP synthesis. **b**) depicts the time course of ΨTP synthesis using 20 mM ΨMP, 5 mM ATP, 60 mM PEP, 1.5 mg/mL CMPK, 0.2 mg/mL PK, 2 mM $MgCl_2$ and 50 mM HEPES pH 8.0 were incubated at 37°C (n =1 individual experiment). c) Depicts the AEX-trace of ΨTP purification using 200 mM NH_4HCO_3 as eluent. For further experimental details and for the analytical procedures used, see the Methods section. Source data are provided as a Source Data file.

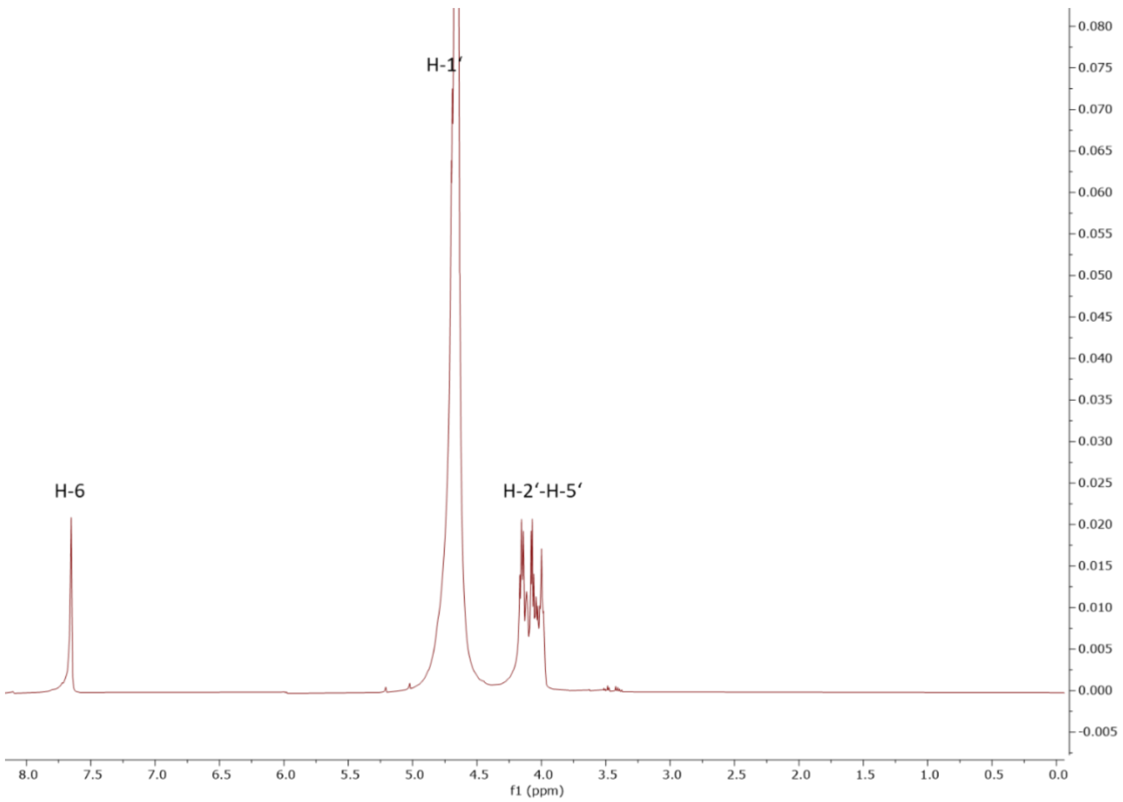

Supplementary Figure 22. ^{1}H NMR of ΨTP (400 MHz, D_2O): δ 7.66 (s, 1H, H-6), 4.72 (d, J = 4.0 Hz, 1H, H-1'), 4.23 – 3.95 (m, 5H, H-2' - H-5').

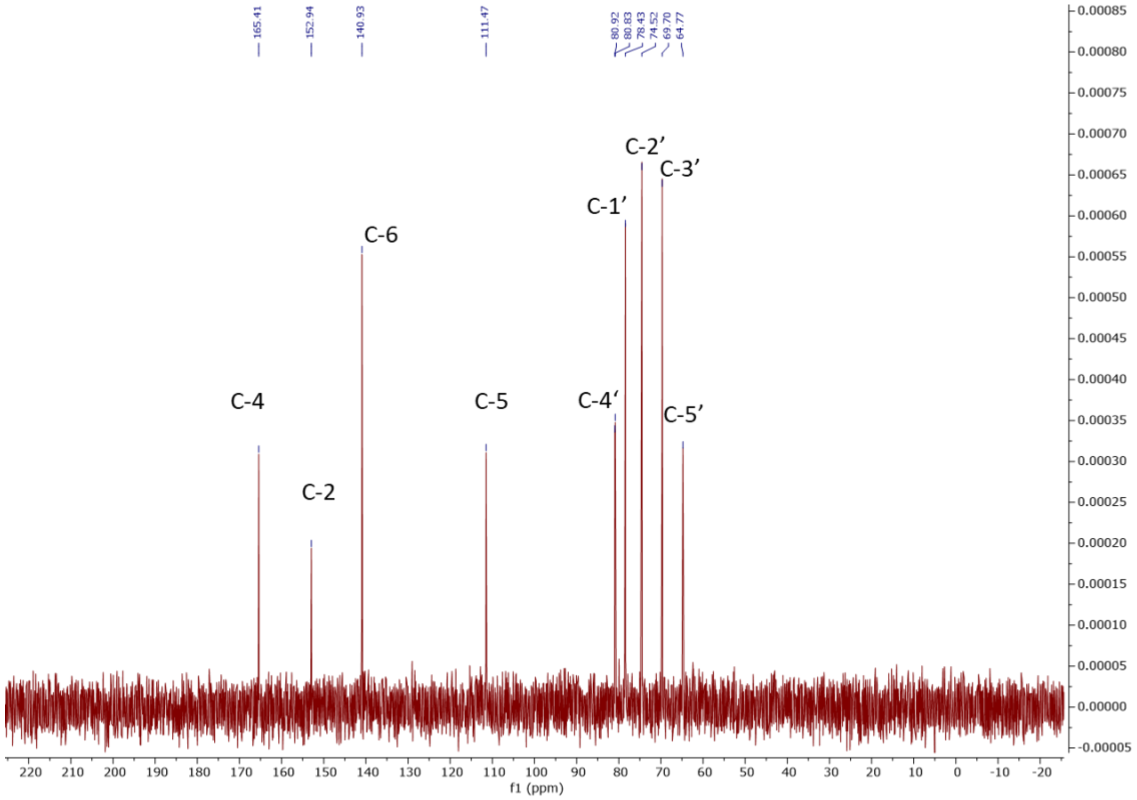

Supplementary Figure 23. ^{13}C NMR of ΨTP (101 MHz, D_2O) δ 165.41 (s, 1C, C-4), 152.94 (s, 1C, C-2), 140.93 (s, 1C, C-6), 111.47(s, 1C, C-5), 80.83 (d, J = 9.0 Hz, 1C, C-4'), 78.43 (s, 1C, C-1'), 74.52 (s, 1C, C-2'), 69.70 (s, 1C, C-3'), 64.77(d, J = 5.5 Hz, 1C, C-5').

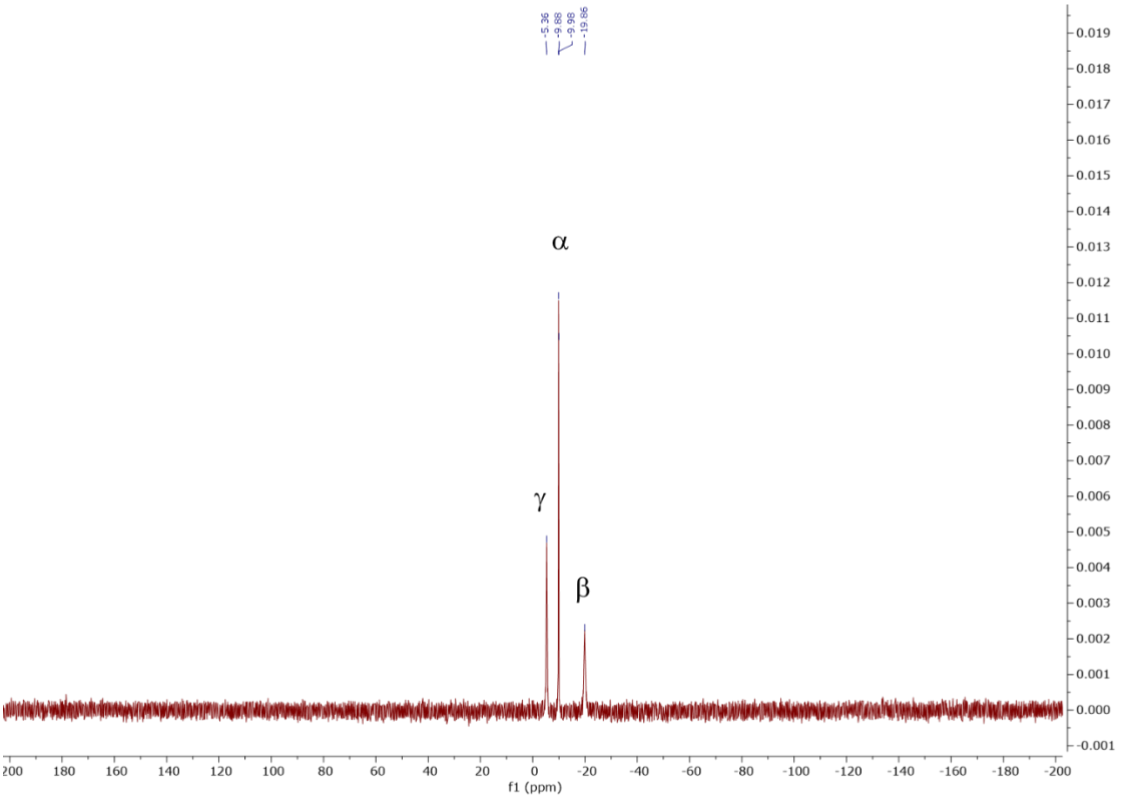

Supplementary Figure 24. ^{31}P NMR of ΨTP (162 MHz, D_2O) δ: -5.36 (1P, γ), -9.93 (d, J = 16.7 Hz, 1P, α), -19.86 (1P, α).

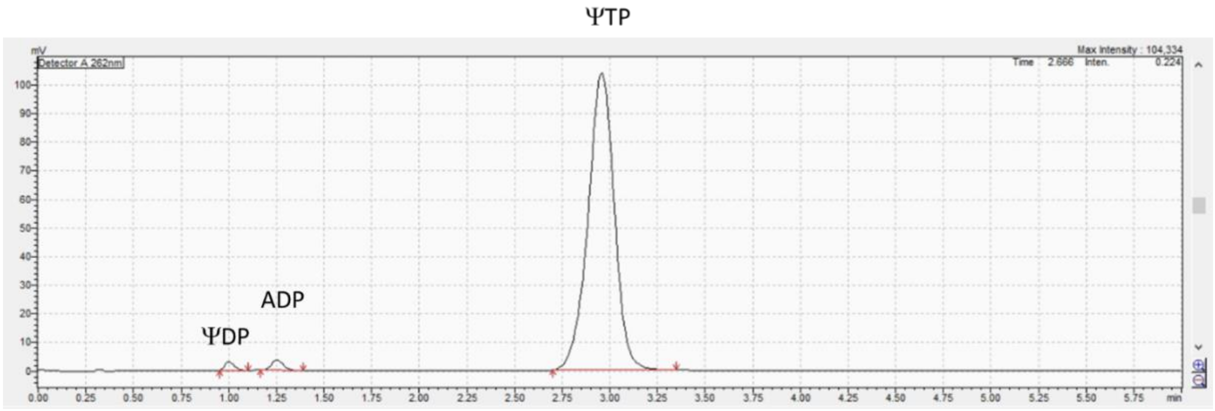

Supplementary Figure 25. HPLC trace of isolated ΨTP.

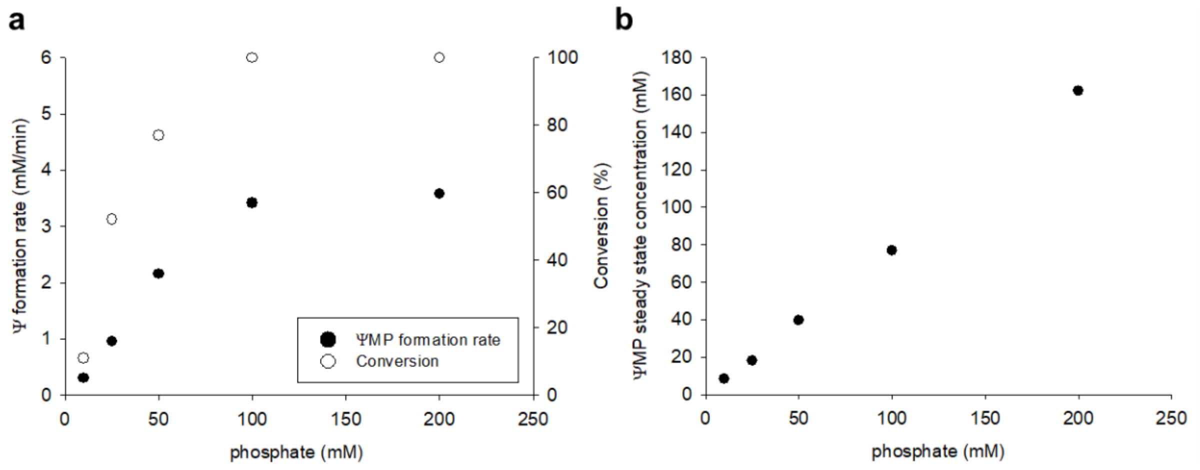

Supplementary Figure 26. The effect of phosphate concentration on the (**a**) Ψ formation rate, conversion and (**b**) ΨMP steady state concentration in the four-enzyme cascade reaction under standard conditions. Reaction conditions: 1.00 M **U**, 10 mM $MnCl_2$, 0.5 mg/mL UP, 5 mg/mL DeoB, 3 mg/mL YeiN and 0.2 mg/mL Yjjg, 30°C, potassium phosphate buffer (pH 7.0) with concentration varied between 10 mM and 250 mM (n =1 individual experiment). For further experimental details and for the analytical procedures used, see the Methods section. Source data are provided as a Source Data file.

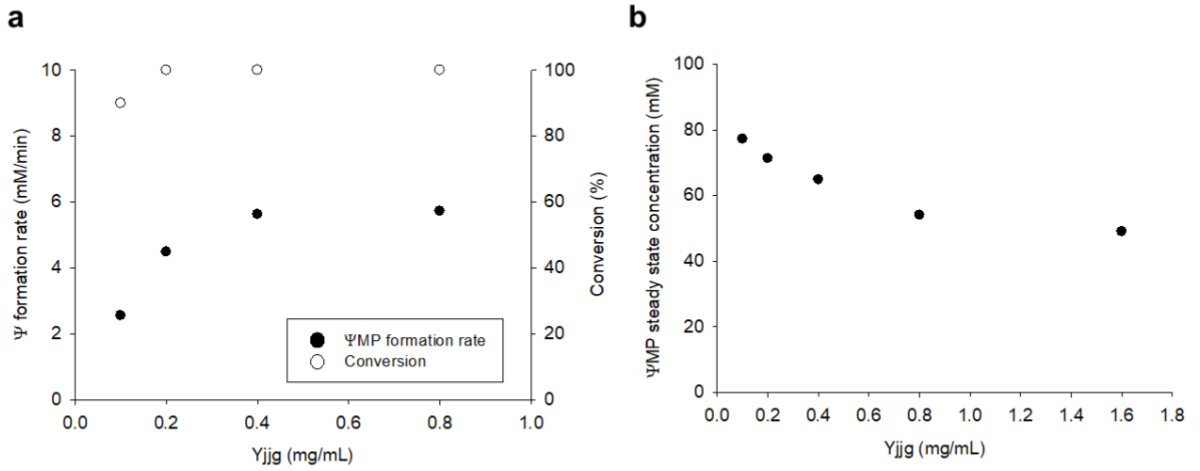

Supplementary Figure 27. Effect of Yjjg loading on the (**a**) Ψ formation rate, conversion and (**b**) ΨMP steady state concentration in the four-enzyme cascade reaction. Reaction conditions: 1.00 M **U**, 10 mM $MnCl_2$, 1 mg/mL 0.5 mg/mL UP, 5 mg/mL DeoB, 3 mg/mL YeiN and 0.1-1.6 mg/mL Yjjg, 30°C, 0.10 M potassium phosphate buffer (pH 7.0) (n =1 individual experiment). For further experimental details and for the analytical procedures used, see the Methods section. Source data are provided as a Source Data file.

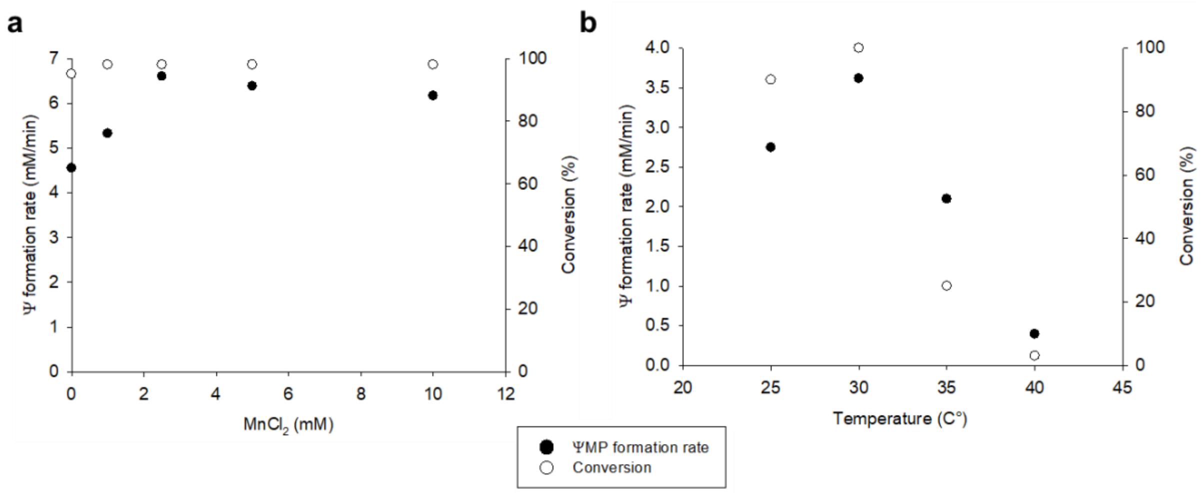

Supplementary Figure 28. The effect of (**a**) $MnCl_2$ concentration and (**b**) temperature on the Ψ formation and conversion in the four-enzyme cascade. Reaction conditions: 1.00 M U, 1-10 mM $MnCl_2$, 0.5 mg/mL UP, 5 mg/mL DeoB, 3 mg/mL YeiN and 0.2 mg/mL Yjjg, 30°C, 0.10 M potassium phosphate buffer (pH 7.0) (n =1 individual experiment). For further experimental details and for the analytical procedures used, see the Methods section. Source data are provided as a Source Data file.

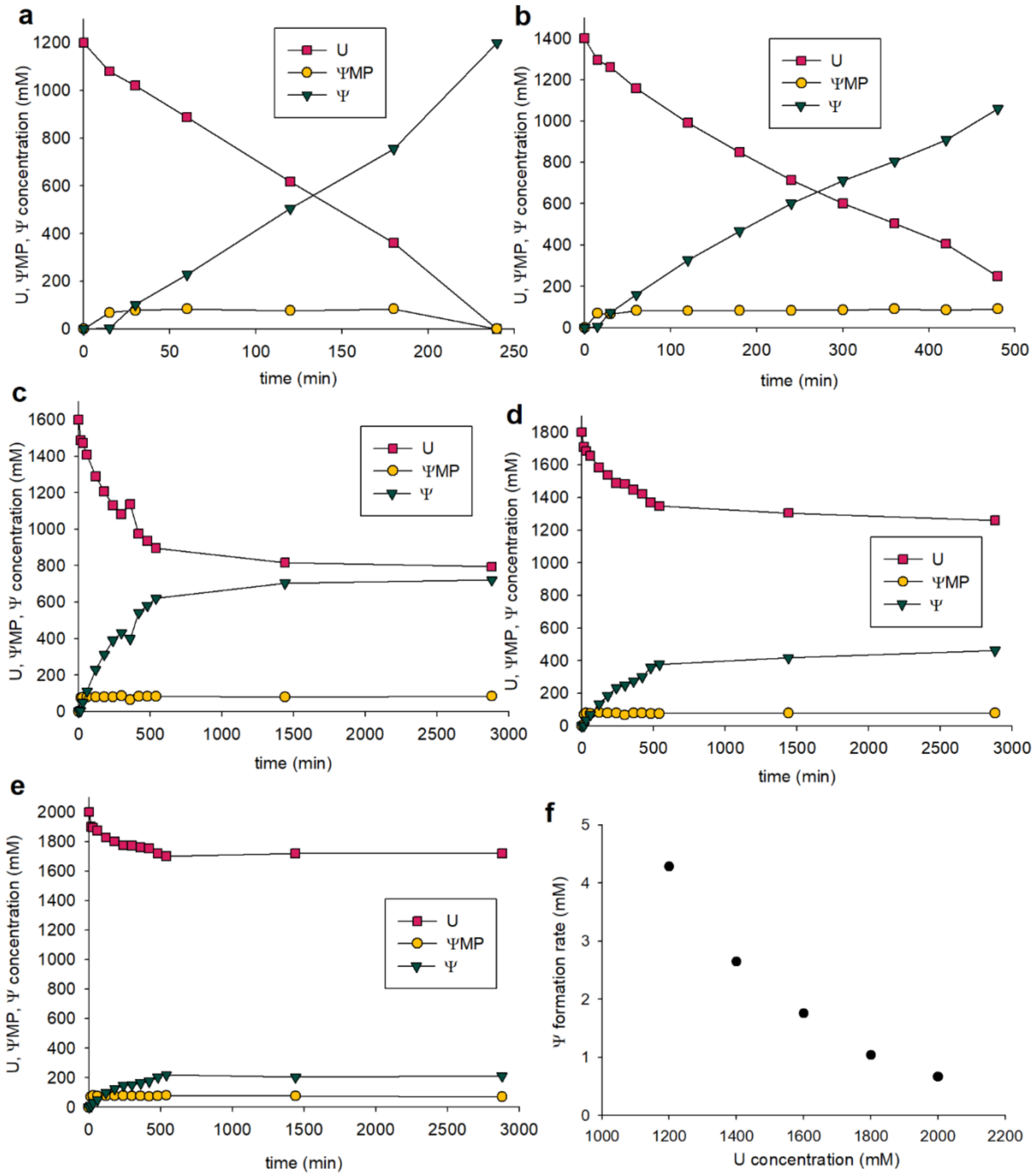

Supplementary Figure 29. Product intensification in the four-enzyme cascade reaction at optimized reaction conditions. (**a**) 1.20 M **U**, (**b**) 1.40 M **U**, (**c**) 1.60 M **U**, (**d**) 1.80 M **U**, (**e**) 2.00 M **U**, (**f**) effect of substrate concentration on the **Ψ** formation rate. Reactions were performed at the indicated **U** concentration in 0.10 M potassium phosphate buffer (pH 7.0), 2.5 mM $MnCl_2$, 0.5 mg/mL UP, 5 mg/mL DeoB, 3 mg/mL YeiN and 0.2 mg/mL Yjjg, at 30 °C (n =1 individual experiment). For further experimental details and for the analytical procedures used, see the Methods section. Source data are provided as a Source Data file.

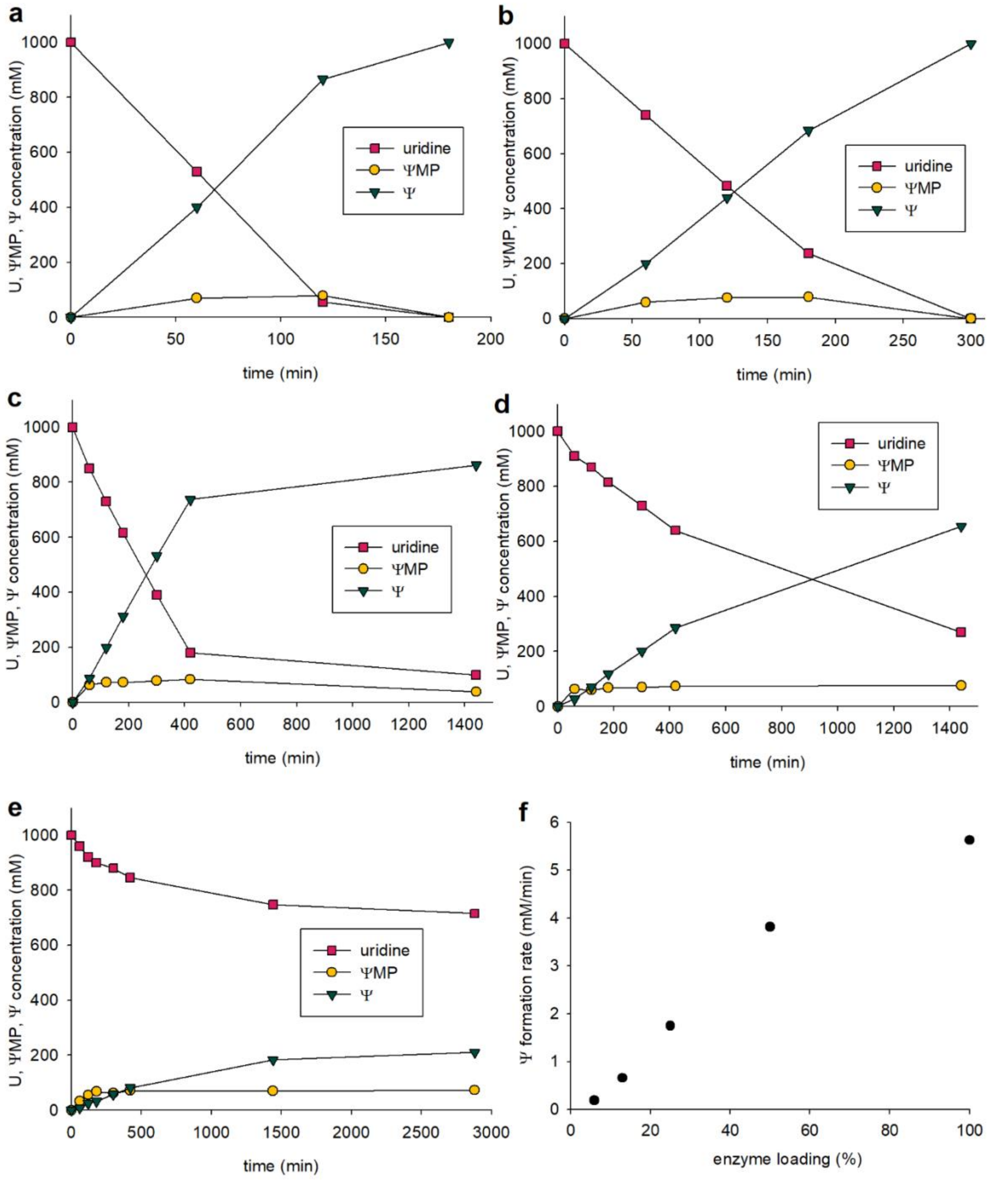

Supplementary Figure 30. Time courses of the four-enzyme cascade reaction with reduced enzyme loading. (**a**) Standard loading: 0.5 mg/mL UP, 5 mg/mL DeoB, 3 mg/mL YeiN and 0.2 mg/mL Yjjg; (**b**) two-fold reduced loading; (**c**) four-fold reduced loading; (**d**) eight-fold reduced loading; and (**e**) sixteen-fold reduced loading. (**f**) The **Ψ** formation rate in dependence of enzyme loading; 100% represents the standard loading. Reactions were performed at 30°C in 0.10 M phosphate buffer (pH 7.0) supplemented with 10 mM $MnCl_2$ and 1.00 M **U** (n =1 individual experiment). For further experimental details and for the analytical procedures used, see the Methods section. Source data are provided as a Source Data file.

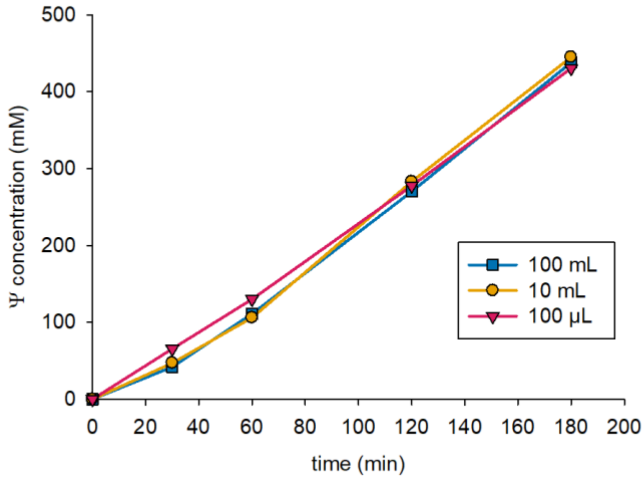

Supplementary Figure 31. Scalability of the four-enzyme cascade reaction. Reactions were performed at 30°C at 100 mL, 10 mL or 100 µL scale using 1.00 M **U** in 0.10 M potassium phosphate buffer (pH 7.0), supplemented with 2.5 mM $MnCl_2$. biocatalyst loading was at 0.3 mg/mL Up, 2.5 mg/mL DeoB, 1.5 mg/mL YeiN and 0.2 mg/mL Yjjg. For further experimental details and for the analytical procedures used, see the Methods section. Source data are provided as a Source Data file. (n =1 individual experiment)

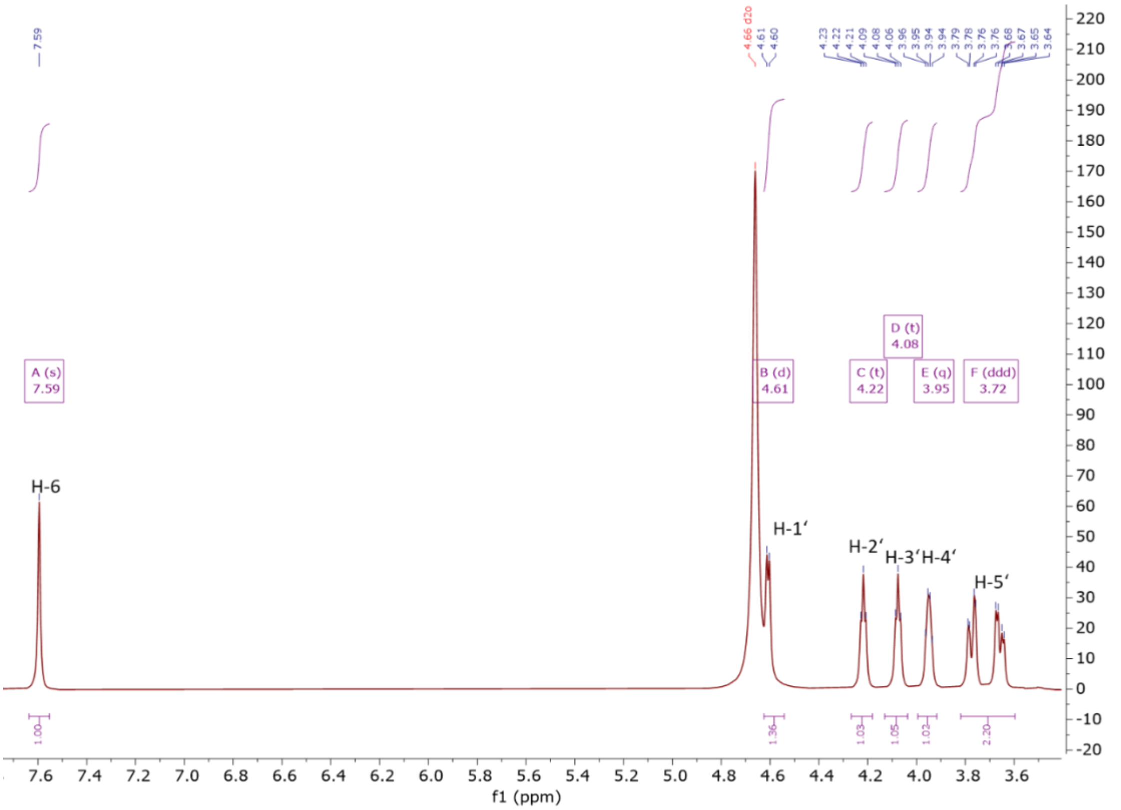

Supplementary Figure 32. ^{1}H NMR of Ψ. (500 MHz, D_2O): δ 7.59 (s, 1H), 4.61 (d, *J* = 5.5 Hz, 1H), 4.22 (t, *J* = 5.4 Hz, 1H), 4.08 (t, *J* = 5.4 Hz, 1H), 3.95 (q, *J* = 4.5 Hz, 1H), 3.72 (ddd, *J* = 58.5, 12.5, 4.0 Hz, 2H).

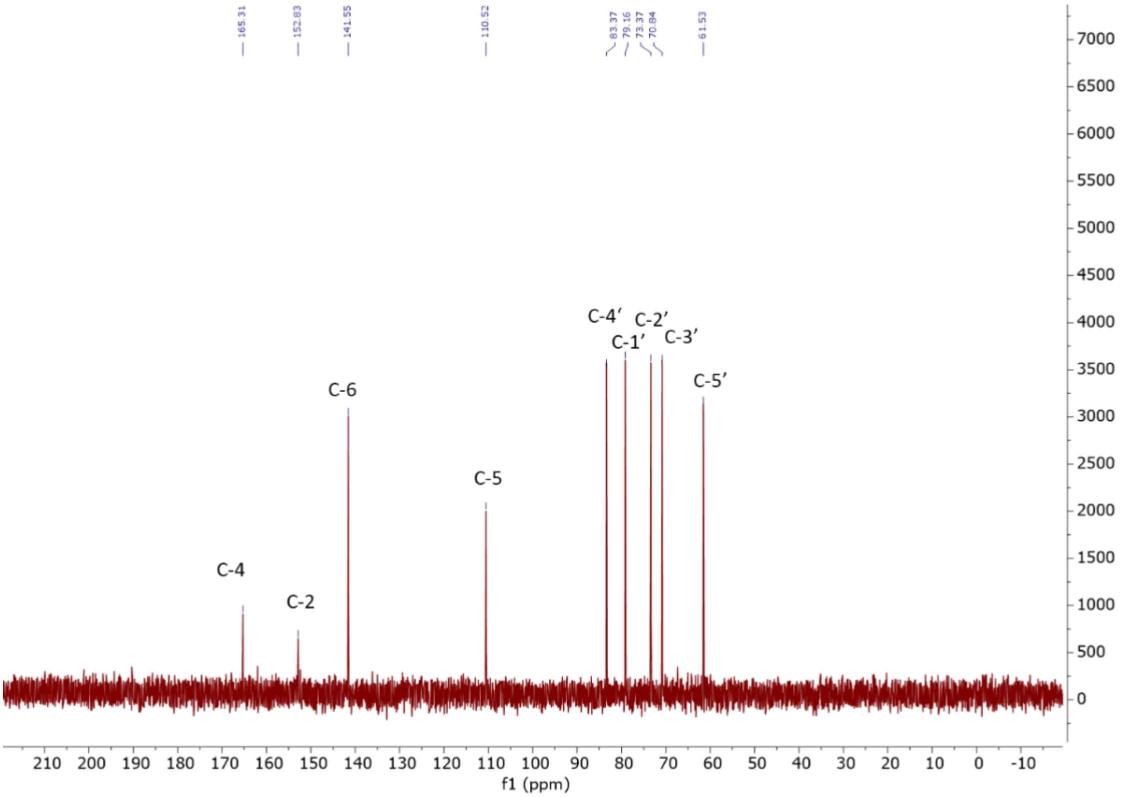

Supplementary Figure 33. ^{13}C NMR of Ψ. (76 MHz, D_2O): δ 165.31, 152.83, 141.55, 110.52, 83.37, 79.16, 73.37, 70.84, 61.53.

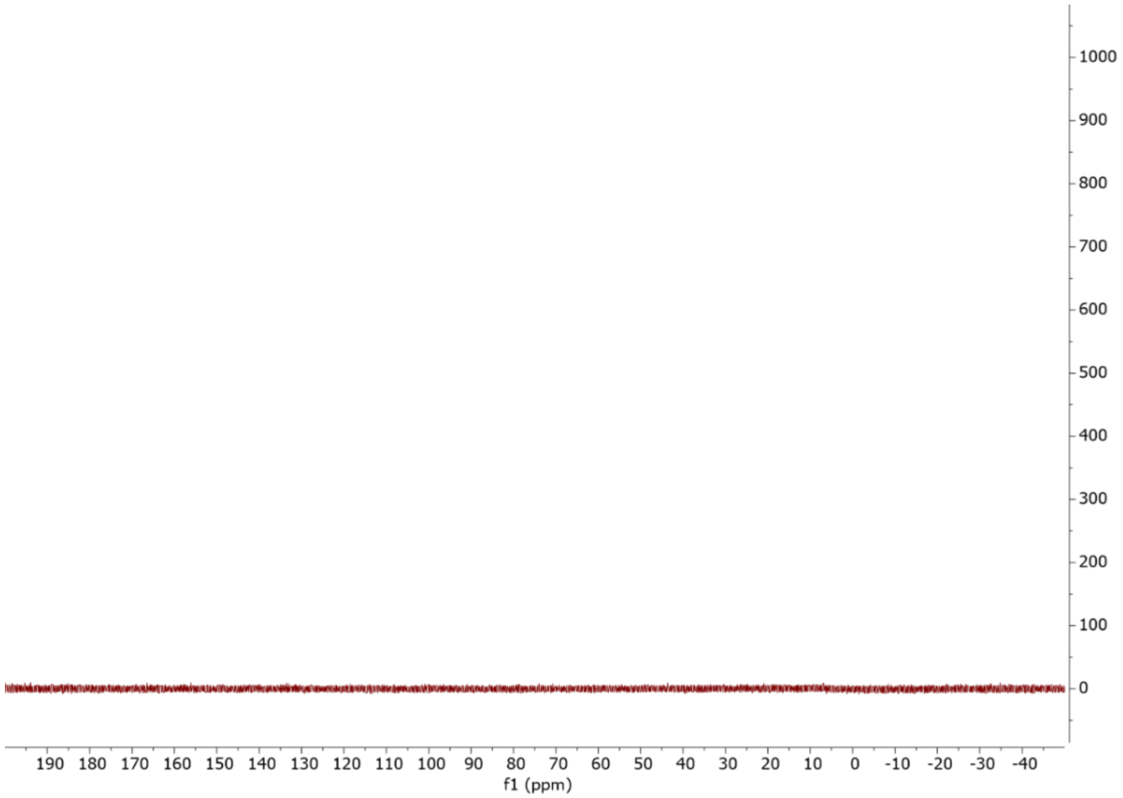

Supplementary Figure 34. ^{31}P NMR of Ψ. (202 MHz, D_2O).

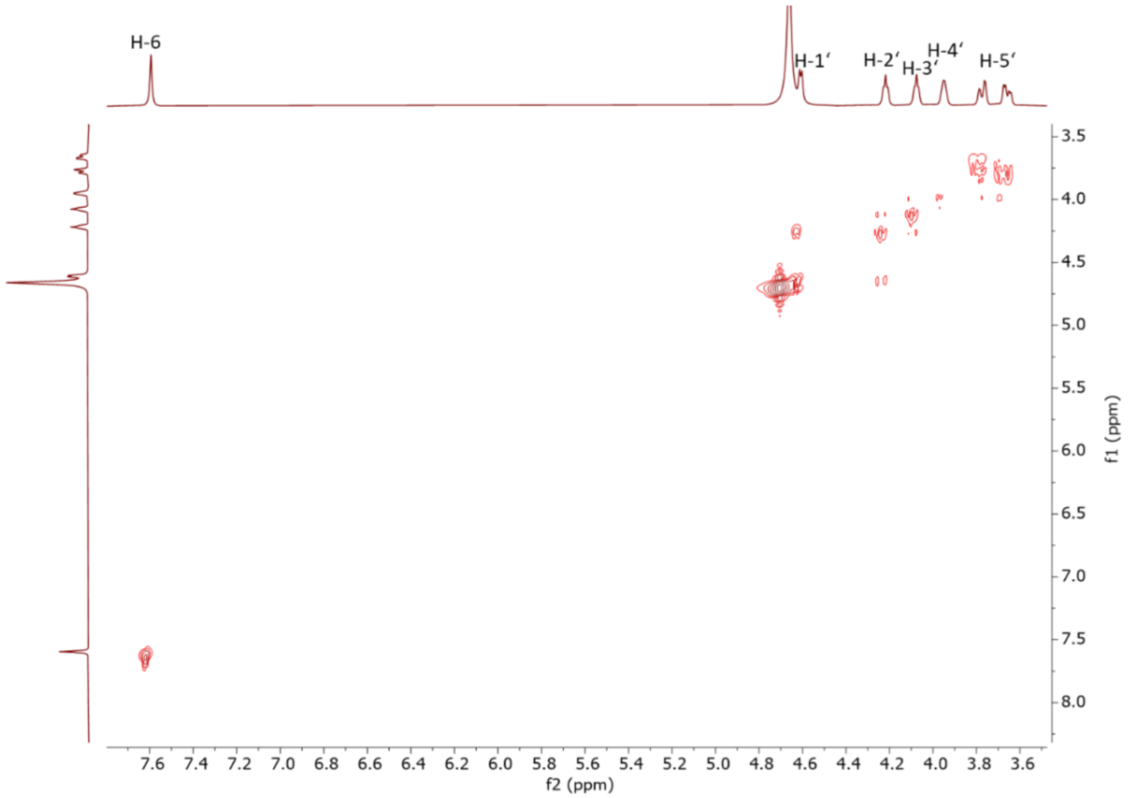

Supplementary Figure 35. COSY-NMR of Ψ. (300 MHz, D_2O) : δ 7.58 (s, H-6, 1H), 4.59 (d, *J* = 5.3 Hz, H-1', 1H), 4.21 (t, *J* = 5.4 Hz, H-2', 1H), 4.06 (t, *J* = 5.3 Hz, H-3', 1H), 3.97 – 3.90 (m, H-4', 1H,), 3.85 – 3.58 (m, H-5', 2H).

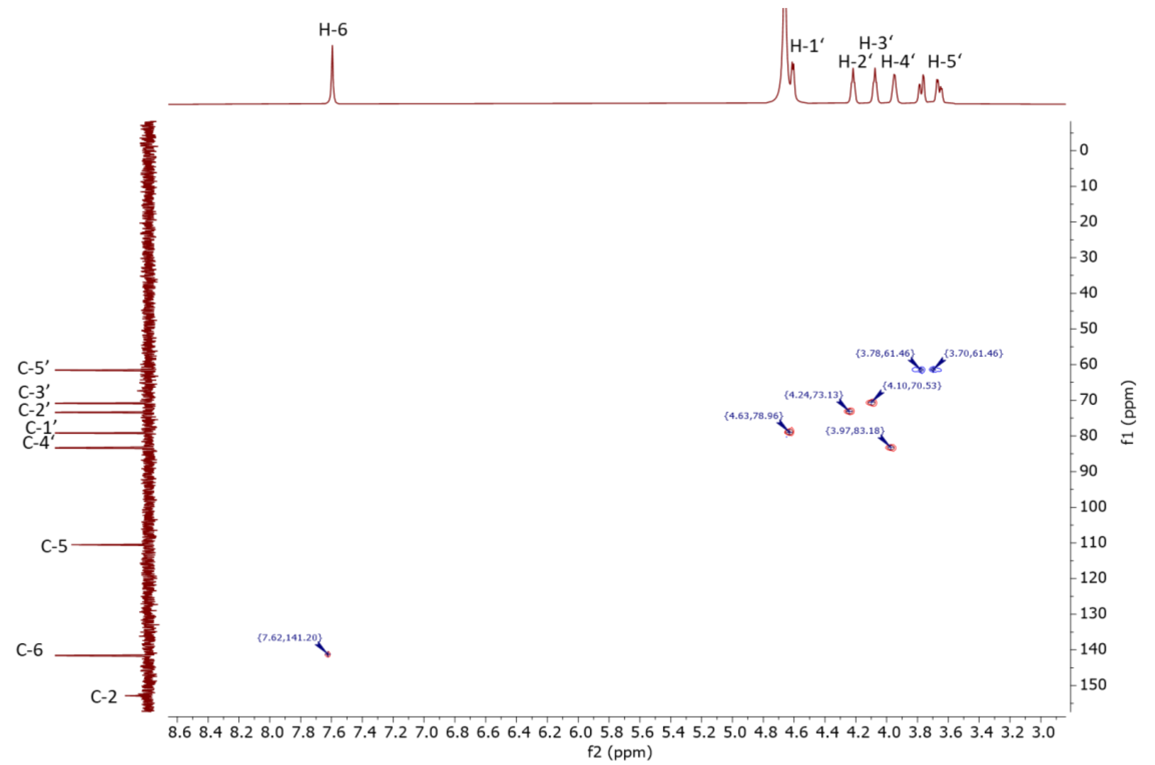

Supplementary Figure 36. HSQC-NMR of Ψ. ^{1}H NMR (300 MHz, D_2O): δ 7.58 (s, H-6, 1H), 4.59 (d, *J* = 5.3 Hz, H-1′, 1H), 4.21 (t, *J* = 5.4 Hz, H-2′, 1H), 4.06 (t, *J* = 5.3 Hz, H-3′, 1H), 3.97 – 3.90 (m, H-4′, 1H,), 3.85 – 3.58 (m, H-5′, 2H); ^{13}C NMR (76 MHz, D_2O): δ 153.58 (C-2), 141.54 (C-6), 110.51 (C-5), 83.37 (C-4′), 79.15 (C-1′), 73.36 (C-2′), 70.83 (C-3′), 61.52 (C-5′).

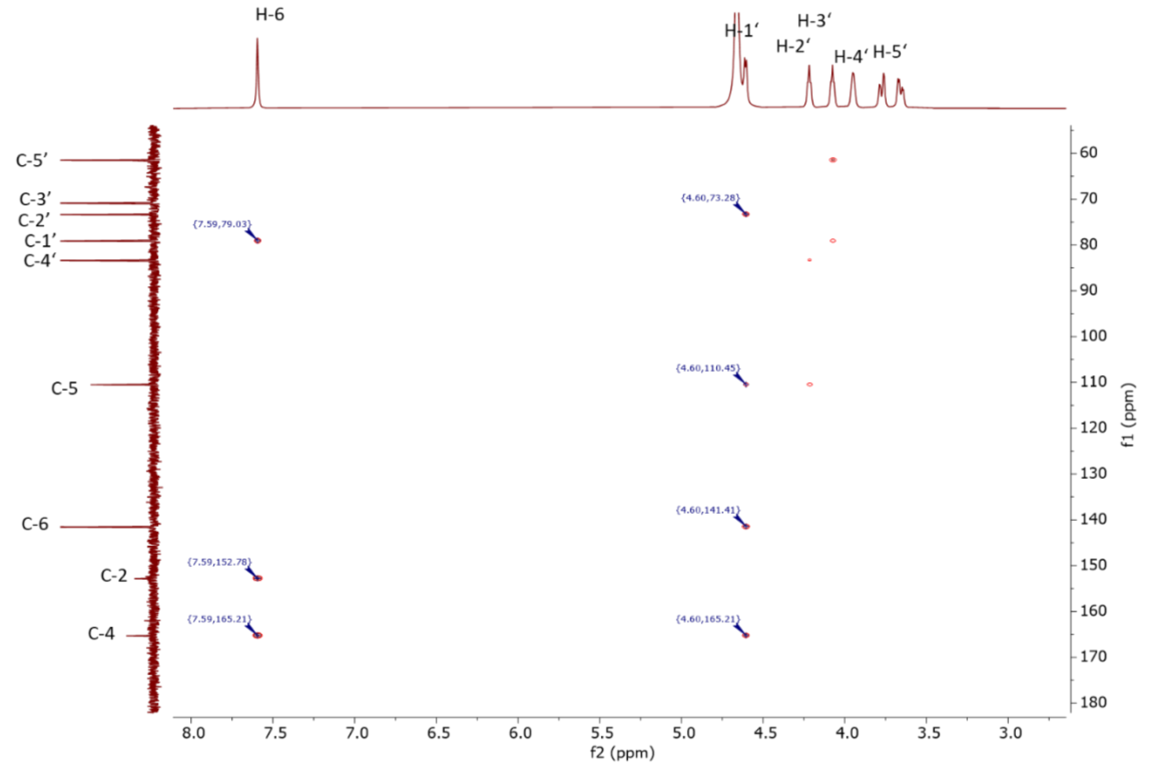

Supplementary Figure 37. HMBC-NMR of Ψ. ^{1}H NMR (300 MHz D_2O): δ 7.58 (s, H-6, 1H), 4.59 (d, *J* = 5.3 Hz, H-1′, 1H), 4.21 (t, *J* = 5.4 Hz, H-2′, 1H), 4.06 (t, *J* = 5.3 Hz, H-3′, 1H), 3.97 – 3.90 (m, H-4′, 1H,), 3.85 – 3.58 (m, H-5′, 2H); ^{13}C NMR (76 MHz, D_2O) : δ 165.31 (C-2), 153.58 (C-4), 141.54 (C-6), 110.51 (C-5), 83.37 (C-4′), 79.15 (C-1′), 73.36 (C-2′), 70.83 (C-3′), 61.52 (C-5′).

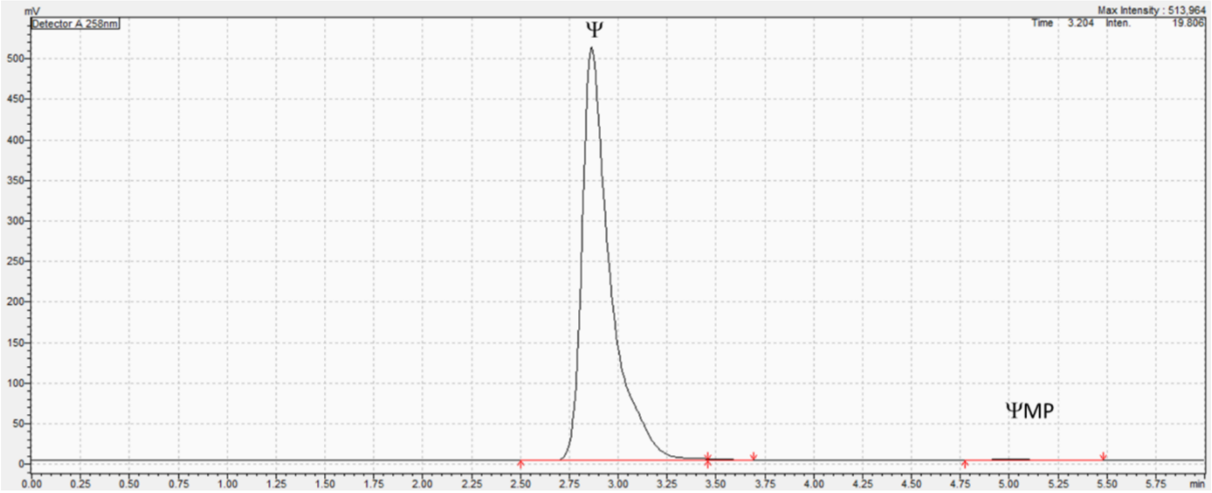

Supplementary Figure 38. HPLC trace of isolated Ψ.

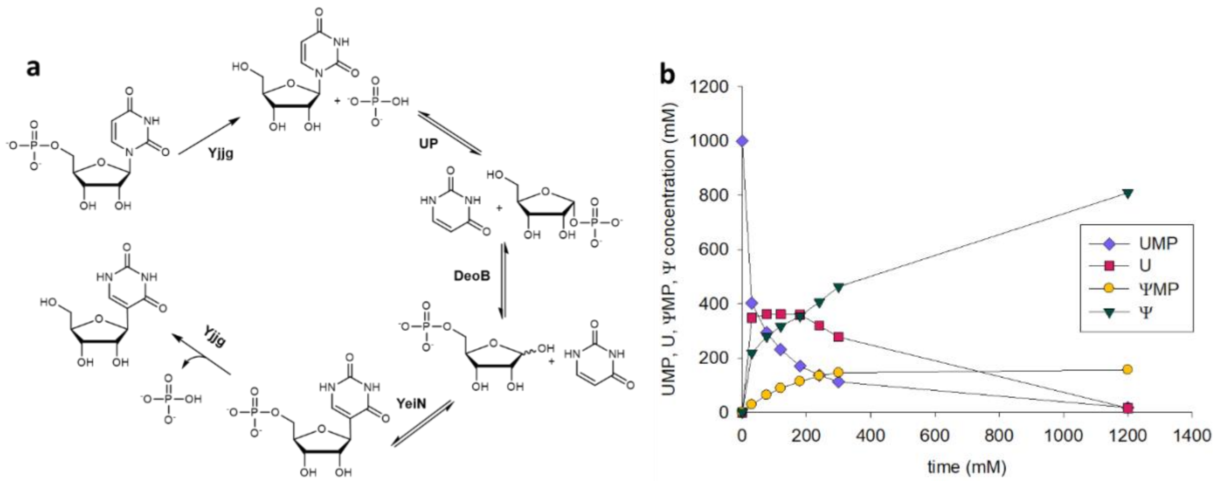

Supplementary Figure 39. Synthesis of Ψ from UMP using the four-enzyme cascade reaction**.** (**a)** Scheme of the enzymatic cascade towards Ψ. (**b)** Reaction time course of Ψ synthesis at 100 µL (25 mg) scale. Reaction conditions: 0.10 M potassium phosphate buffer (pH 7.0), 1.00 M UMP, 10 mM $MnCl_2$, 0.5 mg/mL UP, 5 mg/mL DeoB, 3 mg/mL YeiN and 0.8 mg/mL Yjjg, incubation at 30 °C. For further experimental details and for the analytical procedures used, see the Methods section. Source data are provided as a Source Data file. (n =1 individual experiment)

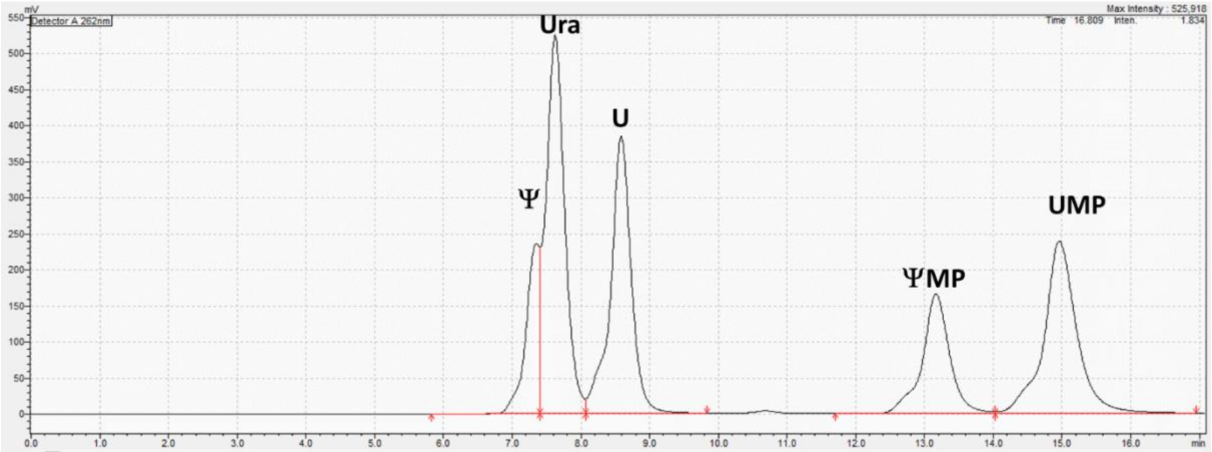

Supplementary Figure 40. HPLC trace of authentic reference substances used and their separation: Ψ (7.3 min), Ura (7.6 min), **U** (8.5 min), ΨMP (13.2 min) and UMP (15.0 min). For further details, see the Methods section.

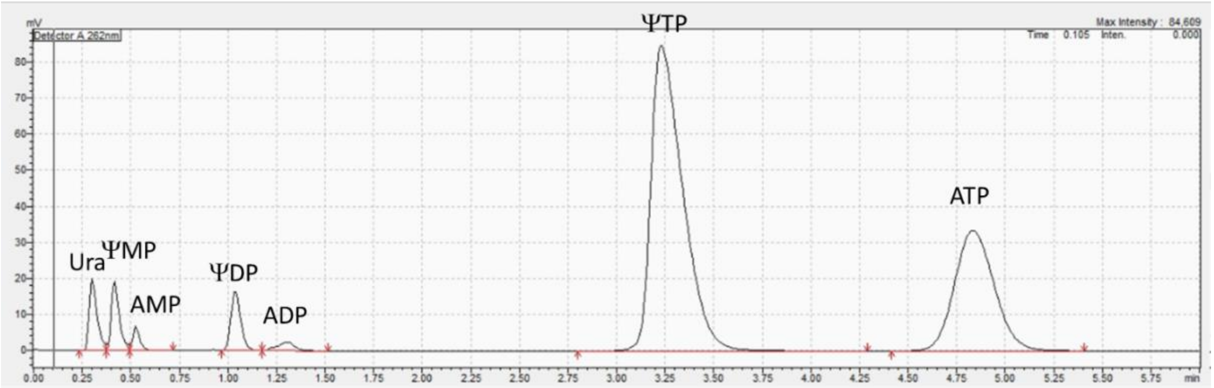

Supplementary Figure 41. HPLC trace of method used for analysis of phosphorylation reactions: Ura (0.3 min), ΨMP (0.4 min), AMP (0.5 min), ΨDP (1.0 min), ADP (1.3 min), ΨTP (3.2 min), and ATP (4.8 min).

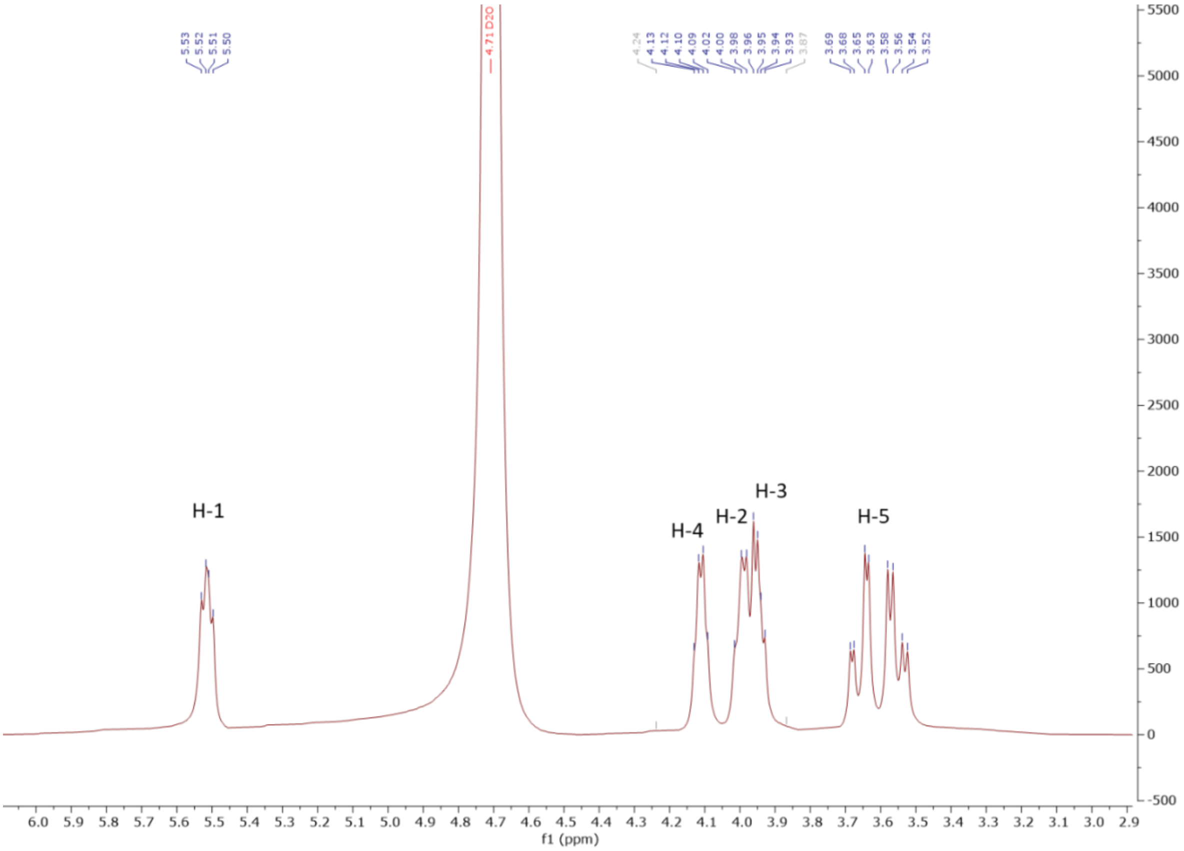

Supplementary Figure 42.^{1}H NMR of Rib1P (300 MHz, D_2O) δ: 5.51 (dd, J = 6.2, 3.9 Hz, 1H, H-1), 4.11 (q, J = 3.8 Hz, 1H, H-4), 4.00 (t, J = 5.1 Hz, 1H, H-2), 3.95 (dd, J = 6.4, 3.6 Hz, 1H, H-3), 3.72 – 3.49 (m, 2H, H-5).
