# Supplementary material for: A selective and atom-economic rearrangement of uridine by cascade biocatalysis for production of pseudouridine
Source: Nat Commun. 2023 Apr 20;14:2261. doi: 10.1038/s41467-023-37942-7 (PMC10116470; doi:10.1038/s41467-023-37942-7)
Supplement: Supplementary file 3 — Reporting Summary [file 41467_2023_37942_MOESM3_ESM.pdf]

nature portfolio

Corresponding author(s): Bernd Nidetzky

Last updated by author(s): Mar 21, 2023

Reporting Summary

Nature Portfolio wishes to improve the reproducibility of the work that we publish. This form provides structure for consistency and transparency in reporting. For further information on Nature Portfolio policies, see our Editorial Policies and the Editorial Policy Checklist.

Statistics

For all statistical analyses, confirm that the following items are present in the figure legend, table legend, main text, or Methods section.

n/a	Confirmed	
☐	☒	The exact sample size (*n*) for each experimental group/condition, given as a discrete number and unit of measurement
☐	☒	A statement on whether measurements were taken from distinct samples or whether the same sample was measured repeatedly
☒	☐	The statistical test(s) used AND whether they are one- or two-sided<br>*Only common tests should be described solely by name; describe more complex techniques in the Methods section.*
☒	☐	A description of all covariates tested
☒	☐	A description of any assumptions or corrections, such as tests of normality and adjustment for multiple comparisons
☐	☒	A full description of the statistical parameters including central tendency (e.g. means) or other basic estimates (e.g. regression coefficient) AND variation (e.g. standard deviation) or associated estimates of uncertainty (e.g. confidence intervals)
☒	☐	For null hypothesis testing, the test statistic (e.g. *F*, *t*, *r*) with confidence intervals, effect sizes, degrees of freedom and *P* value noted<br>*Give P values as exact values whenever suitable.*
☒	☐	For Bayesian analysis, information on the choice of priors and Markov chain Monte Carlo settings
☒	☐	For hierarchical and complex designs, identification of the appropriate level for tests and full reporting of outcomes
☒	☐	Estimates of effect sizes (e.g. Cohen's *d*, Pearson's *r*), indicating how they were calculated

Our web collection on statistics for biologists contains articles on many of the points above.

Software and code

Policy information about availability of computer code

Data collection	Sequences were collected from NCBI, non-redundant protein sequences and nucleotide collection and Unipot. Enzyme parameters were compared to the Brenda database. Molecular mas and extinction coefficents for proteins were calculated using Protparam. eQuilibrator 3.0 was used to estimate the Reaction Gibbs Energy. Lab solution v5.6 from Shimadzu was used for HPLC operation. The VNMRJ v2.2D, Jeol v6.1 or the Bruker Topspin v3.5 software was used for NMR measurements.
Data analysis	Lab solution v5.6 from Shimadzu was used for HPLC data analysis. MestReNova v14.0 was used for NMR analysis. SigmaPlot v10.0 was used for nonlinear fitting of inital rate data. CFX Maestro V. 4.0.2325.0418 was used for evaluation of the Thermal shift assay.

For manuscripts utilizing custom algorithms or software that are central to the research but not yet described in published literature, software must be made available to editors and reviewers. We strongly encourage code deposition in a community repository (e.g. GitHub). See the Nature Portfolio guidelines for submitting code & software for further information.

Data

Policy information about availability of data

All manuscripts must include a data availability statement. This statement should provide the following information, where applicable:

- Accession codes, unique identifiers, or web links for publicly available datasets
- A description of any restrictions on data availability
- For clinical datasets or third party data, please ensure that the statement adheres to our policy

All relevant data are reported in the manusript and in the associated Supplementary Information. All data are available from the corresponding author upon

request. There is no restriction on data availability and Source data are providid within the paper.

Human research participants

Policy information about studies involving human research participants and Sex and Gender in Research.

Reporting on sex and gender	N/A
Population characteristics	N/A
Recruitment	N/A
Ethics oversight	N/A

Note that full information on the approval of the study protocol must also be provided in the manuscript.

Field-specific reporting

Please select the one below that is the best fit for your research. If you are not sure, read the appropriate sections before making your selection.

- [x] Life sciences
- [ ] Behavioural & social sciences
- [ ] Ecological, evolutionary & environmental sciences

For a reference copy of the document with all sections, see nature.com/documents/nr-reporting-summary-flat.pdf

Life sciences study design

All studies must disclose on these points even when the disclosure is negative.

Sample size	The study was performed with purified enzymes. Synthesis reactions and optimizations were typically performed in a single experiment. Kinetics and protein stability measurements were conducted in three individual experiments.<br>The n= employed is the standard for enzyme kinetics and biocatalytic synthesis.
Data exclusions	No data was excluded from the analysis
Replication	Synthesis reactions and optimizations were typically performed in a single experiment. Kinetics and protein stability measurements were conducted in three individual experiments. All attempts of replication were successful, leading to the calculation of consistent kinetic parameters.
Randomization	Randomization is not typically applied in enzyme kinetics determinations/biocatalysis assays.
Blinding	Blinding is not typically applied in enzyme kinetics determinations/biocatalysis assays.

Reporting for specific materials, systems and methods

We require information from authors about some types of materials, experimental systems and methods used in many studies. Here, indicate whether each material, system or method listed is relevant to your study. If you are not sure if a list item applies to your research, read the appropriate section before selecting a response.

Materials & experimental systems

n/a	Involved in the study	
x		Antibodies
x		Eukaryotic cell lines
x		Palaeontology and archaeology
x		Animals and other organisms
x		Clinical data
x		Dual use research of concern

Methods

n/a	Involved in the study	
x		ChIP-seq
x		Flow cytometry
x		MRI-based neuroimaging
